# Viral warfare: unleashing engineered oncolytic viruses to outsmart cancer’s defenses

**DOI:** 10.3389/fimmu.2025.1618751

**Published:** 2025-08-25

**Authors:** Tolulope O. Omolekan, Joy T. Folahan, Mulu Z. Tesfay, Harikrishnan Mohan, Ojasvi Dutta, Leila Rahimian, Khandoker Usran Ferdous, Reza Ghavimi, Aleksandra Cios, Timothy K. Beng, Joseph Francis, Oswald D'Auvergne, Mitesh J. Borad, Konstantin G. Kousoulas, Stephen DiGiuseppe, Bolni Marius Nagalo, Jean Christopher Chamcheu

**Affiliations:** ^1^ Department of Pathological Sciences, School of Veterinary Medicine, Louisiana State University, Baton Rouge, LA, United States; ^2^ Department of Pathology, University of Arkansas for Medical Sciences (UAMS), Little Rock, AR, United States; ^3^ The Winthrop P. Rockefeller Cancer Institute, University of Arkansas for Medical Sciences, Little Rock, AR, United States; ^4^ Department of Pharmacology and Physiology, University of Maryland School of Medicine, Baltimore, MD, United States; ^5^ Marlene and Stewart Greenebaum NCI Comprehensive Cancer Center, University of Maryland School of Medicine, Baltimore, MD, United States; ^6^ Department of Chemistry, Central Washington University, Ellensburg, WA, United States; ^7^ Department of Comparative Biological Sciences, School of Veterinary Medicine, Louisiana State University, Baton Rouge, LA, United States; ^8^ Department of Biological Sciences and Chemistry, College of Sciences and Engineering, Southern University and A&M College, Baton Rouge, LA, United States; ^9^ Department of Molecular Medicine, Mayo Clinic, Rochester, MN, United States; ^10^ Division of Biomedical Sciences, Edward Via College of Osteopathic Medicine, Monroe, LA, United States

**Keywords:** oncolytic viruses, engineered viruses, cancer immunotherapy, combination therapy, immune evasion, pharmacological treatment, tumor targeting, cancer therapy

## Abstract

Oncolytic virotherapy (OVT) has emerged as a promising and innovative cancer treatment strategy that harnesses engineered viruses to selectively infect, replicate within, and destroys malignant cells while sparing healthy tissues. Beyond direct oncolysis, oncolytic viruses (OVs) exploit tumor-specific metabolic, antiviral, and immunological vulnerabilities to reshape the tumor microenvironment (TME) and initiate systemic antitumor immunity. Despite promising results from preclinical and clinical studies, several barriers, including inefficient intratumoral virus delivery, immune clearance, and tumor heterogeneity, continue to limit the therapeutic advantages of OVT as a standalone modality and hindered its clinical success. Recent advances in OV engineering have enhanced viral tropism, immune evasion, and transgene delivery, enabling better tumor targeting and penetration and sustained immune activation in malignant tumors. Moreover, rational combination strategies with immune checkpoint inhibitors (ICIs), chemotherapeutics, and immunometabolic modulators are reshaping OVT into a versatile strategy for precision oncology. This review highlights the mechanistic innovations driving next-generation OV engineering, explores emerging combination regimens, and discusses future directions to overcome resistance and maximize clinical efficacy.

## 1 Introduction

Oncolytic viruses (OVs), both naturally occurring and genetically engineered strains, are emerging as programmable immunotherapeutic that exploit tumor-intrinsic vulnerabilities, such as metabolic rewiring, impaired antiviral defenses, and immune evasion. By leveraging these characteristics, OVs remodel the tumor microenvironment (TME) and elicit systemic antitumor immune immunity (1). Through selective infection and replication, they induce direct oncolysis and immunogenic cell death (ICD), converting immunologically “cold” tumors into “hot” lesions that support T cell infiltration and activation (2).

The concept of oncolytic virotherapy (OVT) dates back to the early 20th-century when spontaneous tumor regressions were observed in patients with leukemia, Burkitt’s lymphoma, and Hodgkin’s disease following viral infections (3). Landmark therapies such as Onyx-015 [a genetically modified adenovirus (AdV)], H101 (AdV variant), and *Talimogene laherparepvec* (T-VEC), a herpes simplex virus-1 [HSV-1 expressing granulocyte-macrophage colony-stimulating factor (GM-CSF)], have since transformed viruses from pathogens into precision-targeted cancer therapeutics (4, 5)

OVs possess either DNA or RNA genomes, which may be single or double stranded. Among them, single-stranded RNA (ssRNA) and double-stranded DNA (dsDNA) viruses are the most commonly used viruses for OV engineering. Notable exceptions include reovirus (a double-stranded RNA virus) and parvovirus (a single-stranded DNA virus). Examples of dsDNA viruses include AdV, vaccinia virus (VacV), and HSV-1. ssRNA viruses are further categorized by polarity into positive-sense (e.g., coxsackievirus, Seneca Valley virus, poliovirus), which are directly translated by host ribosomes, and negative-sense [(e.g., measles virus, Newcastle Disease virus, Vesicular Stomatitis Virus (VSV)], which require transcription into positive-sense RNA before translation. OVs are also classified as either naturally attenuated strains or genetically modified vectors.

OVs may preferentially infect tumor cells due to overexpression of viral entry receptors, disrupted signaling pathways, and impaired antiviral defenses within both tumor cells and the TME. These conditions support viral replication and enable targeted virotherapy across diverse cancer types. Other OVs preferentially target rapidly-dividing, Ras-activated-tumors, IFN-deficient tumors, often of neuroendocrine origin (6). They also differ in their mechanism of egress. For example, HSV-1, AdV, and VacV typically induce rapid lytic egress, while measles and Maraba viruses use non-lytic egress, allowing prolonged survival in the infected cell. Non-lytic egress, is common in enveloped viruses where virions may bud from the plasma membranes or egress via vesicular-mediated pathways.

While not all OVs naturally induce ICD, many can be engineered to do so. Some viruses primarily cause non-immunogenic lysis, whereas others activate immunogenic pathways such as apoptosis, necroptosis, or pyroptosis (7). For example, wild-type AdVs and reoviruses may require genetic modification to trigger ICD effectively. To address this, OVs, notably T-VEC, Maraba virus, VacV and Ad-p53 have been engineered to deliver tumor-suppressor genes, which have been shown to induce hallmark features of ICD, including calreticulin exposure, ATP release, and high mobility group box 1 (HMGB1) secretion (8–10).

Despite their promising potential, OVT faces challenges as a standalone treatment. The TME presents physical and immunological barriers, such as dense extracellular matrix (ECM), hypoxia, and immunosuppressive cytokines [e.g. transforming growth factor-beta (TGF-β) and interleukin-10 (IL-10)], that hinder viral entry, spread, replication, and antitumor immunity (11). Additionally, neutralizing antibodies and tumor heterogeneity further limit efficacy.

To overcome these limitations, next-generation OVs are being actively developed with enhanced delivery systems (e.g. stem cells, nanoparticles, cell-based carriers) and are being combined with other immunotherapies, including ICIs (e.g., anti-PD-1, anti-CTLA-4), chimeric antigen receptor (CAR) T cells, cancer vaccines, radiotherapy, and targeted inhibitors (12–14). OVs are also engineered to express immunomodulatory payloads [e.g. GM-CSF, IL-12, or tumor necrosis factor-related apoptosis-inducing ligand (TRAIL)], incorporate tumor-specific promoters or delete oncogenes (e.g., E1B in AdV, ICP34.5 in HSV-1), to improve safety and selectivity (12–14).

Complementary strategies using epigenetic modifiers (e.g., HDAC inhibitors like valproic acid and vorinostat) and metabolic reprogramming bioactive molecules (e.g., PI3K/Akt/mTOR inhibitors) can sensitize tumors to OV infection (15–17). Nutraceuticals such as curcumin, sulforaphane, and EGCG are also emerging as promising adjuvants that modulate immunity, reverse epigenetic silencing, and enhance viral selectivity (18–20). In this review, we will explore the evolving landscape of OVT, from viral tropism and payload engineering to immunometabolic reprogramming, highlighting its transformative potential in next-generation precision immunotherapy.

## 2 Reprogramming the tumor-OV-immune axis

The TME presents a paradoxical landscape where tumor-driven immune evasion, chronic inflammation, and impaired antiviral signaling support OV replication, while innate immune cells act as key barriers to viral spread. Understanding how OVs navigate and exploit this hostile yet permissive niche is essential for enhancing their efficacy in cancer immunotherapy. OVs reshape the tumor-immune microenvironment by releasing immunostimulatory signals, such as tumor-associated antigens (TAAs), damage-associated molecular patterns (DAMPs), and pathogen-associated molecular patterns (PAMPs), during replication (21). These signals activate pattern recognition receptors (PRRs), including Toll-like receptors (TLRs) and RIG-I-like receptors, on innate immune cells, triggering type I IFN and pro-inflammatory cytokine production. This response promotes local inflammation and recruits antigen-presenting cells (APCs), particularly dendritic cells (DCs), to lymphoid tissues and the TME. Activated DCs capture TAAs and present them to T cells via MHC class I and II pathways, priming CD8^+^ cytotoxic T lymphocytes (CTLs), CD4^+^ helper T cells, and activating natural killer (NK) cells through cytokine signaling and cross-talk with T cells. This cascade drives systemic antitumor immunity while reducing regulatory T cells (Tregs) and myeloid-derived suppressor cells (MDSCs) (22).

To enhance immune activation, engineered OVs are frequently designed to carry bispecific T-cell engagers that boost T-cell priming and cytotoxic function. Additionally, OVs can be modified to express checkpoint inhibitors, pro-inflammatory cytokines, chemokines, costimulatory molecules, or TAAS. Some OVs also activate innate immune pathways like STING or RIG-I, incorporate microRNA target sequences to improve tumor specificity, or disrupt tumor vasculature to facilitate immune infiltration.

Among these strategies, the expression of cytokines and costimulatory ligands plays a critical role in overcoming tumor-induced immune suppression by promoting cytotoxic T lymphocyte (CTL) infiltration and activation. This can convert immunologically “cold” tumors into “hot,” inflamed lesions that are more responsive to immunotherapies, such as checkpoint blockade (22). Collectively, these strategies aim to enhance immune cell recruitment, activation, and tumor-specific responses, often acting synergistically with other forms of immunotherapy. In the following sections, we will examine the innate immune barriers that limit the efficacy of OVT.

### 2.1 Overview of the tumor-OV-immune system triad

The therapeutic outcome of OVT is shaped by the dynamic interplay between the tumor, immune system, and OVs. This triad determines whether OVs are neutralized, persist long enough to cause oncolysis, or successfully trigger systemic antitumor immunity. Upon infection, OVs induce immunogenic cell death (ICD), releasing tumor-associated antigens (TAAs), damage-associated molecular patterns (DAMPs), and pro-inflammatory cytokines that activate antigen-presenting cells (APCs) and prime cytotoxic T lymphocytes (CTLs), initiating abscopal effects that target metastatic lesions (23).

However, this therapeutic potential is often limited by antiviral immunity, tumor heterogeneity, and the immunosuppressive TME (24). Host defenses including PRR-mediated antiviral responses, type I IFN signaling, and neutralizing antibodies can limit OV replication and accelerate their clearance (25, 26). Simultaneously, tumors evade immune detection through MHC downregulation, checkpoint molecule upregulation (e.g., PD-L1, CTLA-4), impaired antigen presentation, and secretion of suppressive cytokines such as TGF-β and IL-10 (27, 28).

Paradoxically, the same immunosuppressive features can make tumors more permissive to OV infection. Many cancer cells harbor defects in antiviral sensing and apoptotic pathways, which help them evade immune surveillance but also create vulnerabilities that OVs can exploit. Factors such as hypoxia, metabolic stress, and acidity further impair IFN signaling, enhancing OV replication, particularly in “cold” tumors like pancreatic ductal adenocarcinoma (PDAC), where VSV and Coxsackievirus A21 thrive (29, 30).

In contrast, healthy cells detect viral infections via pattern recognition receptors (PRRs) such as TLRs and RLRs (31), triggering interferon-stimulated genes (ISGs) and pro-apoptotic pathways that restrict viral spread. Tumor cells often lose this capacity due to mutations or epigenetic silencing in key components like JAK-STAT, PKR, and OAS-RNase L, enabling selective OV replication (31–33). Additionally, overexpression of viral entry receptors [(e.g., Coxsackievirus and Adenovirus Receptor (CAR), nectin-1, EGFR)] enhances OV tropism (34). For example, glioblastoma and lung tumors with high CAR expression are particularly susceptible to AdV-based OVs, while PDAC with impaired IFN signaling supports VSV and Coxsackievirus A21 infection (34).

Navigating this triad requires balancing viral persistence and immune activation. While innate sensing and IFN responses are critical for systemic antitumor immunity, they can also prematurely eliminate therapeutic viruses. To overcome these barriers, next-generation OVs are engineered to evade or modulate immune detection, resist antiviral pathways, and express immunostimulatory payloads. These strategies convert “cold” tumors into “hot,” inflamed lesions that recruit and activate effector immune cells. Combination approaches further enhance efficacy by transiently suppressing innate antiviral defenses during OV administration and subsequently reactivating immune responses to promote tumor clearance (35–37).

### 2.2 TME creates immune suppression to favor OV infection

From the earliest stages of malignancy, cancer cells establish an immunosuppressive TME characterized by chronic inflammation, cell exhaustion and weak immunogenicity. While these conditions hinder immune surveillance, they paradoxically support selective OV infection and replication, and localized immune activation (38). As discussed in Section 2.1, the tumor-OV-immune triad shapes therapeutic outcomes. Here, we focus specifically on how the immunosuppressive TME and its inherent heterogeneity modulates to OV permissiveness and influences the outcome of OVT.

**Figure 1:**
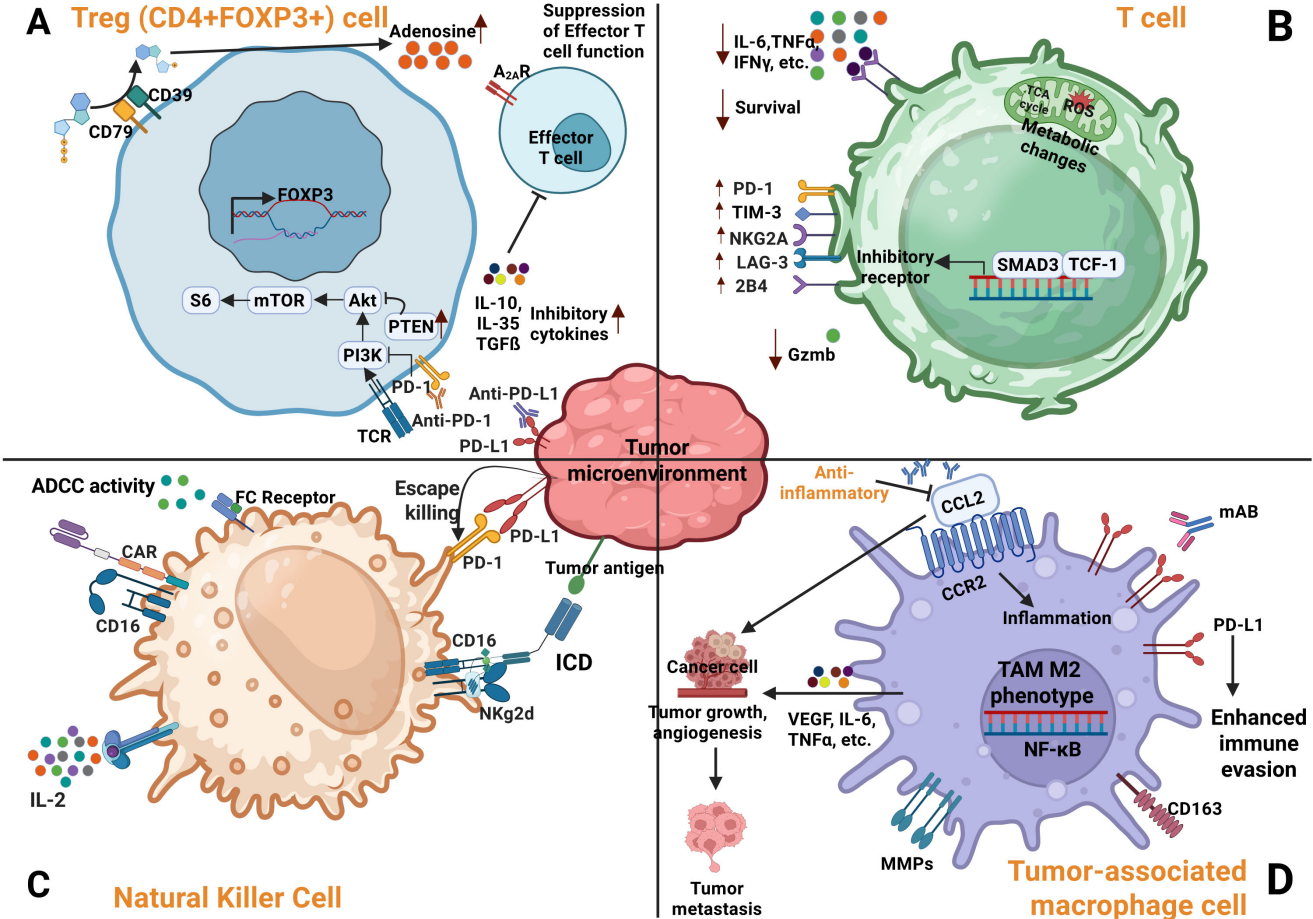
Regulation of immune cells activity within the TME. **(A)** Suppression of regulatory cells (Tregs) in the TME by anti PD-1/PD-L1 therapy leads to inhibition of PI3K signaling pathways and increased adenosine production. **(B)** T cell exhaustion within the TME is characterized by decreased cytokine production and elevated expression of inhibitory receptors. **(C)** Tumor cells evade NK cell mediated cytotoxicity through strong interactions between NK cell inhibitory receptors and their ligands expressed on cancer cells. **(D)** Immune evasion by cancer cells is further enhanced by TAMs, which contribute to angiogenesis, tumor growth, and metastasis (38).

Tumors heterogeneity further influences immune engagement, giving rise to distinct immune phenotypes, immune-inflamed, immune-excluded, and immune-desert, each presenting unique challenges and opportunities for OVT (38). The TME contains immunosuppressive stromal cells, such as cancer-associated fibroblasts (CAFs), lymphatic endothelial cells as well as infiltrating immunosuppressive cells, such as MDSCs, regulator T cells (Tregs), and tumor-associated macrophages (TAMs), all of which restrict CTL infiltration and function. CAFs secrete immunosuppressive cytokines (e.g., TGF-β, IL-10) and upregulate checkpoint molecules (e.g., PD-L1 and CTLA-4) (**Figure 1**), reinforcing immune escape. Although they express MHC-II and can present antigens to CD4^+^ T cells, their dominant role in the TME is suppressive. MHC-II expression is regulated by IFN-γ via Janus kinase/signal transducers and activators of transcription (JAK-STAT) pathway and the class II trans-activator (CIITA) (39). Lymphatic endothelial cells, while facilitating immune cell trafficking, also promote Treg expansion and local immune tolerance. These suppressive features impair IFN signaling and antiviral responses, creating metabolic stress that facilitates OV propagation. Engineered OVs expressing ICIs (e.g., anti-PD-1, anti-CTLA-4) or immunostimulatory cytokines (e.g., GM-CSF) can counteract these signals, reinvigorate T-cell function, and enhance both viral oncolysis and immune-mediated tumor clearance (40, 41).

PD-L1 [also known as cluster of different (CD274) or B7 homology 1 (B7-H1)], expressed on APCs, macrophages, and tumor cells bind PD-1 on T cells, recruiting SHP-1 and SHP-2 phosphatases. This inhibits T cell receptor (TCR) signaling by dephosphorylating CD3 and Zeta-chain-associated protein kinase 70 (ZAP70) and suppress IL-2 secretion (42). This cascade not only blunts T-cell activation but also promotes regulatory T cell (Treg) differentiation and polarization of TAMs toward the immunosuppressive M2 phenotype. These M2-TAMs secrete pro-tumorigenic cytokines such as vascular endothelia growth factor (VEGF), fibroblast growth factor, and TNF-α, supporting angiogenesis and metastasis (43). PD-1 expression on NK cells also suppresses their cytotoxic activity, further aiding immune evasion.

CTLA-4, another key checkpoint, competes with CD28 for B7 binding, limiting T-cell co-stimulation and metabolic fitness. It also impairs APC function and nutrient uptake. LAG-3 exacerbates T-cell dysfunction by disrupting calcium signaling during TCR activation (44–46). In preclinical models, deletion of PD-1 or SHP-2 enhances antigen presentation and T-cell activation, underscoring the therapeutic potential of targeting these pathways to restore antitumor immunity.

In the TME, various cell types, including tumor cells, CAFs, TAMs, endothelial cells, and DCs, overexpress viral entry receptors, which can be exploited for OVT. Tumor cells frequently upregulate receptors such as CAR, heparan sulfate proteoglycans, and EGFR, due to oncogenic mutations or loss of tumor suppressors like p53 and RB. These changes are driven by tumor-intrinsic signaling, inflammatory cues, and epigenetic regulation. Similarly, CAFs express neuropilin-1 (NRP1) and integrins; TAMs may express DC-SIGN and ACE2; endothelial cells lining tumor vasculature express ACE2, NRP1, and integrins; and APCs like DCs express DC-SIGN (CD209) and L-SIGN, collectively enhancing OV tropism and entry (47–51).

Mutations in p53 and RB further shape the TME. p53 loss promotes immune evasion by downregulating antigen presentation, increasing secretion of immunosuppressive cytokines, and recruiting Tregs and MDSCs (52). It also weakens ISG expression in chemotherapy-resistant tumors like GBM, enabling oHSV-1 replication (53). RB inactivation, common in HPV-associated cancers, enhances E2F activity and S-phase entry, creating conditions favorable for replication of S-phase-dependent OVs like AdVs and HSV (53, 54). Overexpression of anti-apoptotic proteins such as Bcl-2 also impairs OV-induced cell death, but this can be countered by engineering OVs to express pro-apoptotic transgenes or inhibitors of survival signaling pathways in tumor cells, thereby enhancing therapeutic oncolysis (55).

Chronic inflammation remodels the TME, promotes recruitment of suppressive population (Tregs, MDSCs, TAMs), and enhances OV infection by weakening antiviral defenses (56–60). Elevated cytokines such as TNF-α and IL-6 increase vascular permeability, facilitating OV delivery, but also inhibit immune cell function and upregulate immune checkpoints, further dampening antitumor immunity (56, 57).

Overall, the immunosuppressive, metabolically dysregulated, and chronically inflamed TME, while a barrier to immune surveillance, offers a strategic opportunity for OVT. By targeting impaired antiviral sensing, overexpressed viral entry receptors, and metabolic vulnerabilities, rationally engineered OVs can achieve enhanced selectivity and therapeutic efficacy. As our understanding of these mechanisms deepens, next-generation OVs are being designed with greater precision, reinforcing their role in the evolving landscape of cancer immunotherapy.

### 2.3 Overcoming immune barriers to optimize OVT

**Figure 2:**
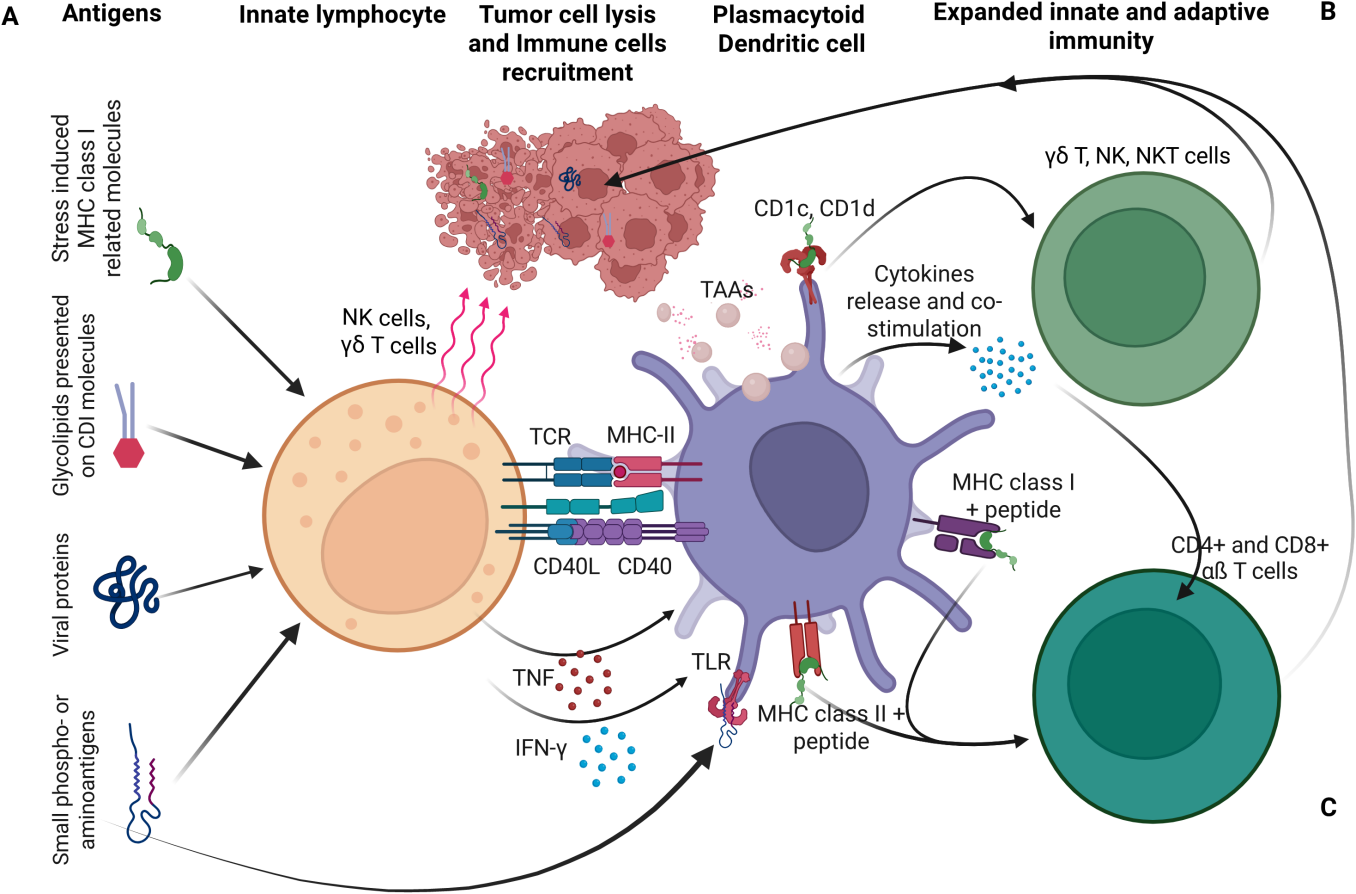
Dendritic cells (DCs) as a central link between innate and the adaptive immunity. **(A)** Innate lymphocytes, including T cells, NKT cells, and NK cells recognize pathogen-derived or self-antigens on infected cells, transform, or stressed cells. Their activation promotes DC maturation, particularly when DCs present activating ligands recognized by these lymphocytes. **(B)** Activated DCs in turn amplify innate immune responses. **(C)** DCs also stimulate adaptive immunity by processing and presenting antigens, including those from lysed cells, to naïve T cells. Cytokines and cell contact-dependent molecules interactions mediate DC activation by various innate lymphocytes. In turn, DC-derived cytokines support further expansion and differentiation of both innate and adaptive immune cells (61).

DCs play a central role by capturing tumor antigens released during OV-induced ICD. DAMPs and proinflammatory cytokines promote DC maturation, especially when combined with type I IFNs or innate agonists like STING or TLR ligands. Mature DCs migrate to lymph nodes, present antigens to naïve T cells, and initiate CTL responses limiting viral persistence. Plasmacytoid dendritic cells (pDCs), found in blood, lymphoid organs, and bone marrow, are specialized antiviral sentinels that detect viral RNA and CpG DNA via TLR7 and TLR9 (**Figure 2**). Upon activation, they secrete large quantities of type I IFNs that suppress OV replication and activate CTLs (61, 62). In addition to IFN production, DCs migrate to lymph nodes, present viral antigens via MHC I and II, and initiate adaptive immune responses by activating CD4^+^ helper T cells, CD8^+^ CTLs, and B cells, leading to antibody production and virus-specific cytotoxicity.

While these responses are essential for controlling natural infections, they can limit OV persistence and therapeutic efficacy, especially after systemic administration. To address this, recent strategies aim to transiently suppress pDC activation or reprogram their function, either to extend the window for OV replication or to harness their antigen-presenting capacity to enhance systemic antitumor immunity (63, 64). Other innate immune cells also restrict OV efficacy. NK cells rapidly recognize virus-infected tumor cells via stress ligands and missing-self signals, releasing cytotoxic granules and IFN-γ to recruit additional immune effectors (65). Macrophages, including Kupffer cells and splenic macrophages, efficiently clear circulating viral particles, while tissue-resident macrophages release antiviral cytokines like TNF-α and type I IFNs. Neutrophils further restrict viral spread through reactive oxygen species and neutrophil extracellular traps (66). The complement system adds another layer of defense. Activation of classical and alternative pathways leads to opsonization and lysis of viral particles, particularly in patients with pre-existing antiviral antibodies, posing a challenge for intravenous OV delivery (67).

Additional innate effectors, such as conventional dendritic cells (cDCs) and natural killer T (NKT) cells, bridge innate and adaptive immunity. cDCs detect viral PAMPs via TLRs and RLRs, produce type I IFNs and pro-inflammatory cytokines, and present antigens to T cells. NKT cells recognize lipid antigens via CD1d and amplify early cytokine responses that restrict viral propagation (68). Central to these defenses is type I IFN signaling. IFN-α/β binding activates the JAK-STAT pathway, inducing antiviral effectors like PKR, OAS-RNase L, and Mx proteins that degrade viral RNA, inhibit protein synthesis, and create a hostile environment for viral replication (69). While these mechanisms protect against opportunistic infections, they also limit the therapeutic window for OVs.

To overcome these barriers, innovative strategies are being developed, including transient immunosuppression, polymeric shielding of viral particles, and the use of carrier cells to deliver OVs to tumors. Balancing these approaches, by exploiting tumor-intrinsic vulnerabilities while fine-tuning host innate responses, offers a promising path to improving the durability and potency of oncolytic virotherapy (63, 64).

Despite the immune system’s capacity to detect and eliminate pathogens, many viruses have evolved sophisticated mechanisms to evade immune surveillance. These natural strategies form the basis for engineering OVs that can persist within tumors, avoid premature immune neutralization, and enhance therapeutic efficacy. RNA viruses like VSV, measles virus, and Newcastle Disease Virus naturally mutate their surface proteins to escape antibody recognition (70). Engineered OVs mimic this adaptability through immune cloaking strategies such as PEGylation, CD47 coating, or envelopment in extracellular vesicles. For example, CD47-coated AdVs, extracellular vesicle-shielded VacV, and measles virus with mutated envelope proteins evade phagocytic clearance and prolong persistence in the TME (71).

Critically, many tumors exhibit defects in innate antiviral defenses, particularly in the 2′-5′-OAS-RNase L and PKR pathways, key components of type I IFN-mediated responses. These sensors detect viral double-stranded RNA (dsRNA) and initiate RNA degradation, translational arrest, or apoptosis to suppress viral replication (72, 73). However, dysfunction in these pathways allows OVs to evade immune detection and selectively replicate in malignant cells (73, 74).

To exploit this IFN-related vulnerabilities, introduced in section 2.1., OVs have evolved mechanisms to inhibit these antiviral sensors. Some encode proteins that directly block RNase L (e.g., HSV-1’s ICP34.5 and SKIV2L), while others produce decoy RNAs that bind OAS, preventing the synthesis of 2′-5′-linked oligoadenylates required for RNase L activation. Structural modifications in viral RNA can also reduce recognition by OAS, and some OVs minimize dsRNA production to avoid triggering both OAS-RNase L and PKR pathways. For instance, VacV uses E3 and K3 proteins to inhibit OAS and PKR, AdVs deploy VA RNA to block PKR activation, and Newcastle Disease Virus expresses a V protein that prevents PKR recognition of viral RNA (75–78). These adaptations enhance OV replication, tumor lysis, and immune activation, particularly in immune-cold tumors where antiviral defenses are already suppressed (79, 80). Tumors with impaired IFN signaling and MHC downregulation are particularly permissive to viruses like HSV and cytomegalovirus, which naturally evade immune detection. This principle has guided the development of engineered HSV strains (e.g., G207, G47Δ) with deletions in ICP34.5, enabling selective replication in IFN-deficient tumor cells while sparing healthy tissue (79, 81). Some OVs are further engineered to transiently suppress antigen presentation, delaying immune clearance and enhancing intratumoral replication (81).

## 3 Strategies to enhance OVs for tumor selectivity and potency

Reengineered as programmable immunotherapeutic agents, OVs can seamlessly breach complex tumor defenses with precision. Leveraging next-generation strategies, such as enhanced tumor tropism, improved intratumoral penetration, and immune-metabolic reprogramming, they mount a coordinated, multifaceted assault on cancer cells. When combined with checkpoint inhibitors, small molecule, radiotherapy, and metabolic modulators, OVT transcend monotherapy limitations and emerged as a cornerstone of multimodal cancer therapy.

### 3.1 Genetic manipulation strategies

#### 3.1.1 Through directed evolution to enhance tumor selectivity and potency

Directed evolution emerged as a powerful strategy for optimizing OVs in the early 2000s, following its broader application in molecular biology and protein engineering in the 1990s. This iterative approach accelerates the natural selection of viral variants with enhanced tumor specificity, immune evasion, and replication efficiency in cancer cells. By subjecting large populations of recombinant precursor viruses to rounds of mutagenesis, selection, and amplification under tumor-like conditions, researchers have generated highly selective and potent oncolytic candidates with broad therapeutic potential. Unlike rational genetic engineering, which involves targeted modifications based on known viral mechanisms, directed evolution enables the discovery of superior tumor-killing variants while preserving safety and minimizing off-target effects (82).

A landmark application of directed evolution in OV development involved the optimization of HSV, VacV, and AdVs, where viral strains were subjected to selective pressures that enhanced their oncolytic potency. By the mid-to-late 2000s, this approach gained wider adoption in OV research, leading to significant advancements in viral adaptation. For instance, directed evolution was applied to engineered reoviruses and VacV capable of replicating more efficiently conditions characteristic of TME and aggressive cancers (83, 84). Among the most notable successes, ColoAd1, a chimeric AdV (Ad11p/Ad3), was developed through this process, demonstrating superior tumor selectivity. This virus exhibited up to 100-fold higher replication in human colon tumor tissues compared to normal tissues, underscoring the potential of directed evolution in generating highly effective oncolytic agents (85).

Further applications of this strategy in reovirus engineering resulted in variants with mutations in the λ2 and σ1 proteins, enhancing their infectivity and oncolytic efficacy in preclinical melanoma models (86). To expand host range, some reovirus variants evolved in Junctional Adhesion Molecule-A (JAM-A)-deficient cell lines, acquiring mutations in the σ1 and μ1 proteins that enabled binding to sialic acid coreceptors, thereby improving infection efficiency in a broader spectrum of cancer cells (87). Additional adapted variants exhibited enhanced tumor cell binding and apoptosis induction, though safety concerns remained (87). Efforts to refine IFN sensitivity led to reovirus variants that discriminated more effectively between malignant and normal cells, improving safety profiles. Similarly, attenuating JAM-A receptor binding yielded viruses with reduced pathogenicity but sustained oncolytic potential in preclinical models (82). Most of these modifications targeted the σ1 protein, which governs reovirus attachment to cellular receptors, highlighting the importance of fine-tuning viral entry mechanisms to balance infectivity and safety (88).

Despite its promise, directed evolution presents challenges. The emergence of unpredictable mutations poses a risk of generating variants with unintended pathogenicity or increased replication in normal cells. The selection process itself is labor-intensive and requires precise design of selective pressures and screening methodologies. Moreover, balancing multiple desirable traits, such as enhanced infectivity, robust oncolytic activity, and safety, remains a complex undertaking. While directed evolution has yielded promising preclinical results, translating these advances to human clinical applications is complicated by factors such as host immune responses, viral clearance, and the influence of the TME on viral efficacy (83). Nonetheless, the continued refinement of directed evolution strategies holds significant potential for developing next-generation OVs with improved therapeutic outcomes.

#### 3.1.2 Through gene deleting and editing techniques to attenuate pathogenicity

A key challenge in OV engineering is achieving a balance between potent oncolytic efficacy and host safety. Attenuation strategies, particularly gene deletion and genome editing have emerged as key tools to restrict viral replication to tumor cells while minimizing off-target effects in normal tissues. Gene deletions are often designed to disable viral genes essential for replication in healthy cells but redundant in cancer cells, thereby enhancing tumor specificity. For instance, deletion of thymidine kinase (TK) gene in HSV restricts replication to rapidly dividing tumor cells while sparing normal cells (89). In T-VEC, deletions of γ34.5 and α47 gene combined with insertion of GM-CSF gene, enhances tumor selectivity and immune activation (55). Similarly, ONYX-015, an engineered AdV lacking E1B-55kDa gene, preferentially replicates in p53-deficient cancer cells (90). Deletion of the TK gene in JX-594/Pexa-Vec, an engineered VacV, likewise restricts replication to proliferative cancer cells, reducing systemic toxicity (55). Innovative synthetic chimeric viruses, such as vesiculovirus, incorporating Morreton virus glycoprotein with VSV genes, have shown promising safety and immunogenicity profiles in Ewing sarcoma and fibrosarcoma models, inducing potent CD8^+^ T-cell responses and tumor regression (91). Engineered strains like VSV-IFNβ and VSV- MΔ51-IFNβ encode IFNβ, to enhance immune stimulation while maintaining controlled replication and safety (92).

More recently, CRISPR-Cas9 technology has revolutionized OV genome engineering by enabling precise deletions, insertions, and modifications. In large-genome viruses such as HSV, AdV, and VACV, CRISPR has streamlined the process of creating recombinant constructs. In HSV-1, both non-homologous ends joining (NHEJ) and homology-directed repair (HDR) pathways have been used for gene knockouts and knock-ins in a single step (93). In VacV, CRISPR/Cas9 has facilitated simultaneous knockout of immunosuppressive genes (e.g., N1L, A46R) and insertion of TAA (e.g., TRP2), effectively transforming the virus into a therapeutic vaccine (94). In adenoviral systems, CRISPR-induced indels have shown tumor-selective replication and heritability.

#### 3.1.3 Tumor-selective replication via miRNA and promoter engineering

miRNAs are key regulators of tumor progression, invasion, and immune evasion. Their dysregulation in cancer cells provide a unique opportunity for miRNA-guided OV selectivity. By incorporating miRNA response elements (MREs) into viral genomes, OVs can be programmed to replicate preferentially in cancer cells while being suppressed in normal tissues. For instance, Coxsackievirus B3 modified with MREs for miR-1, miR-216 (high in normal tissues), and miR-143, miR-145 (low in tumors) achieves selective replication and potent antitumor activity in breast cancer and melanoma models (95, 96). This selective targeting ensures preferential viral replication in cancer cells, minimizing off-target effects. When combined with melittin (a lytic peptide) and CpG oligodeoxynucleotides (TLR9 agonists), these modified viruses demonstrate potent antitumor activity in breast cancer and melanoma models (96).

Additionally, OVs can deliver tumor-suppressive miRNAs such as miR-143, which downregulates oncogenes like K-RAS and induces apoptosis. This has been demonstrated in both AdV and VSV platforms in colorectal and osteosarcoma models (97, 98). Tumor-specific miRNAs like miR-21 and miR-222 have also been exploited to regulate viral replication through engineered binding sites, ensuring selective activation in cancer cells (99, 100). For example, incorporation of miR-21-binding sites into the 3′ untranslated region of the UL9 gene allow oncogenic miR-21 (overexpressed in most cancers) to reactivate viral replication selectively in tumor cells (99). Similarly, miR-222, a key factor in viral propagation, has been targeted using oAdVs modified with miR-222-binding sites, sensitizing tumor cells to oncolysis (100). Complementing miRNA strategies, tumor-specific promoters (e.g., hTERT, PSA, alpha-fetoprotein, mucin-1, Oct4, Nanog, or Sox2) have been integrated into essential viral genes of OVs (like those for replication or lysis) to restrict gene expression to cancer cells, further refining selectivity (101).

Promoter engineering complements microRNA (miRNA) targeting strategies in a synergistic way. While promoter engineering ensures positive selectivity, activating viral replication only in CSCs, miRNA targeting provides negative selectivity by preventing replication in normal cells. This is achieved by inserting miRNA response elements (MREs) into the viral genome that are recognized by miRNAs highly expressed in normal tissues but downregulated in CSCs. When the virus enters a normal cell, these miRNAs bind to the MREs and suppress viral gene expression, effectively silencing replication. In contrast, in CSCs where these miRNAs are absent or low, the virus can replicate freely.

Promoter engineering is a powerful strategy in OVT and it involves modifying the viral genome so that key genes required for replication are placed under the control of CSC-specific promoters. These promoters are only active in cells expressing certain transcription factors, commonly found in CSCs, ensuring that the virus replicates selectively in malignant cells while sparing normal tissue. Promoters such as Oct4, Nanog, Sox2, and Nestin are frequently used because they are highly active in CSCs but largely inactive in normal differentiated cells.

Together, promoter engineering and miRNA targeting create a dual-layered safety and specificity system. This combination ensures that OVs are both activated in the right cells (via promoters) and inhibited in the wrong ones (via miRNAs), making them highly precise tools for targeting CSCs in solid tumors. Also, they have significantly enhanced OV specificity, reduced pathogenicity, and paved the way for safer, more effective OVTs. As precision genome editing continues to evolve, these tools are poised to accelerate the clinical translation of next-generation OVs (91).

### 3.2 Cancer stem cells therapeutic targeting

#### 3.2.1 Receptor targeting

Successful OVT hinges on precise targeting of cancer stem cells (CSCs), which are known to drive tumor recurrence, metastasis, and resistance to conventional therapies, ensuring the selective destruction of cancer cells while sparing normal cells. This specificity is achieved through a combination of natural viral tropism, genetic engineering, and strategic exploitation of the TME (102).

CSCs often express unique or overexpressed surface receptors that distinguish them from normal stem cells and differentiated tumor cells. Some viruses naturally infect cancer cells due to the overexpression of specific receptors, while others can be engineered to exploit these differences, allowing for selective infection and destruction of CSCs. HSV-1, AdV, measles virus, and CVA21 exploit receptors such as nectin-1/HVEM, Coxsackievirus and Adenovirus Receptor (CAR), CD46/EGFR, and ICAM-1 to infect melanoma, epithelial, ovarian, and bladder cancers (103, 104). Reovirus, preferentially infects cells with activated Ras signaling through the junctional adhesion molecule-A (JAM-A) while vaccine-strain measles virus (MV-Edm) infect tumors overexpressing CD46, like GBM, lymphomas, and certain carcinomas, impairing the tumor-initiating capacity of their CSCs and enhancing their sensitivity to chemotherapy (105). CD133 is a well-established marker found on CSCs in glioblastoma, colorectal, and liver cancers. CD133-targeted oncolytic measles virus (MV-CD133) selectively target CSCs expressing CD133, helping prevent tumor reoccurrence (106). EpCAM, another surface marker prevalent in epithelial-derived CSCs, has also been used as a target for OV-mediated therapy. Additionally, integrins such as αvβ3 and αvβ5, which are upregulated in CSCs and tumor vasculature, have been exploited by AdVs and measles viruses to improve tumor selectivity and viral spread.

To enhance receptor targeting, OVs can be genetically modified through techniques such as pseudotyping, where viral envelope proteins are replaced with those from other viruses that naturally bind CSC-specific receptors. Ligand insertion is another approach, where peptides or single-chain antibodies (scFvs) are inserted into viral capsid proteins to direct binding to CSC markers.

These strategies collectively increase the specificity of OVs, reduce off-target effects, and enhance therapeutic efficacy. Moreover, targeting CSCs with OVs not only eliminates the root of tumor resistance but also promotes the release of TAAs, thereby stimulating a robust antitumor immune response. This makes receptor targeting a powerful and promising approach in the development of OV-based cancer therapies.

#### 3.2.2 Suicide genes engineering

A major advancement in OVT is the integration of suicide genes, which enhance both therapeutic efficacy and safety by promoting tumor cell destruction while modulating the TME. These genetic modifications exploit cancer-specific vulnerabilities, such as defective antiviral responses and dysregulated signaling pathways, to achieve precise tumor targeting. Suicide genes function by encoding enzymes that convert non-toxic prodrugs into cytotoxic agents, selectively eliminating infected tumor cells. Among the most extensively studied is HSV-TK, which phosphorylates ganciclovir into a toxic nucleotide analog, leading to DNA chain termination and apoptosis. Another well-characterized enzyme is cytosine deaminase, which metabolizes 5-fluorocytosine into 5-fluorouracil, a potent inhibitor of DNA synthesis. Additionally, nitroreductase activates the prodrug CB1954, which induces DNA cross-linking and widespread tumor cell death (107).

Integrating suicide genes into OVs offers multiple therapeutic advantages. By combining viral oncolysis with prodrug activation, this approach enhances tumor specificity while minimizing systemic toxicity. The controlled administration of prodrugs enables spatiotemporal regulation of cytotoxicity, ensuring that tumor destruction remains highly targeted. A particularly valuable feature of suicide gene therapy is the bystander effect, in which toxic metabolites diffuse into adjacent cancer cells, amplifying the therapeutic impact beyond directly infected cells (108, 109).

Beyond direct tumor lysis, suicide gene-induced apoptosis also contributes to antitumor immune activation. The ICD triggered by these mechanisms enhances the recruitment and activation of immune cells, promoting a sustained systemic antitumor response. This dual approach, combining localized viral replication with systemic immune engagement, positions suicide gene therapy as a powerful strategy for durable tumor suppression (109). OVs expressing suicide genes are currently under extensive preclinical and clinical evaluation. HSV-1-based OVs encoding HSV-TK have shown promising results in GBM models, while AdVs and VacV engineered with suicide genes are being investigated for various solid malignancies. However, challenges remain, including optimization of delivery, enhancement of tumor specificity, and overcoming immune-mediated viral clearance. Advances in genetic engineering, such as combining OVs with ICIs, radiotherapy, and chemotherapy, may further enhance therapeutic efficacy, bringing suicide gene-armed OVs closer to widespread clinical application (110).

Clinically, these targeting strategies pave the way for personalized OVT, enabling combination with chemotherapy, radiation, and immunotherapy. Beyond precision, OVs can overcome resistance by lysing refractory tumor cells and exposing novel antigens, initiating broader immune responses (40). While immune clearance and delivery remain challenges, nanoparticle-based systems and carrier cell technologies are rapidly advancing toward clinical application.

## 4 Synergistic strategies to overcome TME barriers and boost OVT efficacy

### 4.1 Metabolic reprogramming

OVs represent a promising strategy to enhance T cell function and metabolism, particularly in tumors that are resistant to conventional therapies. By reshaping the metabolic landscape of T cells, OVs contribute to sustained antitumor immune responses and amplify the efficacy of existing immunotherapies (111).

A key mechanism involves metabolic reprogramming of tumor-infiltrating T cells. OVs interact with glycolytic enzymes in infected cells, increasing energy availability to support T cell expansion and function (112). Additionally, OV-induced ICD and release of tumor antigens and DAMPs activate and metabolically reprogram T cells to sustain a potent antitumor immune response (113). Some OVs are engineered to express metabolic regulators, such as leptin, to further enhance T cell fitness and tumor eradication (21).

OVs also improves T cell infiltration and activation within the TME. By disrupting immunosuppressive barriers, they enhance the trafficking and function of CAR T cells and endogenous T cells (114). When combined with immune checkpoint inhibitors (ICIs) or adoptive T cell therapies, OVs create synergistic effects that overcome the limitations of monotherapies and promote durable antitumor responses (102). Engineered OVs frequently express cytokines such as IL-2, IL-12, and TNF-α, which are essential for T cell proliferation, survival, and metabolic activation (115). IL-2 promotes clonal expansion, IL-12 enhances cytotoxicity, and TNF-α increases tumor vasculature permeability, facilitating deeper immune infiltration. These cytokines also stimulate glycolysis and oxidative phosphorylation, supporting sustained CTL activity.

In parallel, OVs enhance antigen presentation by upregulating MHC molecules and recruiting DCs, which prime naïve T cells and reinforce metabolic reprogramming. This coordinated activation equips T cells with the energy and signaling support needed for long-term tumor control (41, 116). This effect is further strengthened by DC recruitment and activation, leading to increased T cell priming and metabolic reprogramming, equipping T cells with the necessary energy reserves to sustain a long-term antitumor response.

To further amplify T cell responses, OVs can be engineered to express costimulatory molecules such as OX40L, CD40L, CD137, ICAM-1, and GITR (117–120). For example, VALO-D102, an AdV encoding CD40L and OX40L, induces robust CD8^+^ T cell infiltration and tumor control in melanoma models, with enhanced efficacy when combined with anti-PD-1 therapy (119). Similarly, LOAd703, encoding CD40L and CD137, activates cytotoxic T cells and upregulates immunostimulatory molecules (CD80, CD86, CD70), MHC, and adhesion molecules like ICAM-1, improving tumor immunogenicity and therapeutic outcomes in multiple myeloma (120).

By integrating oncolysis, metabolic support, antigen presentation, costimulatory signaling, and checkpoint inhibition, OVs offer a powerful strategy for enhancing T cell-based cancer immunotherapy. These synergistic mechanisms collectively drive more effective and durable antitumor responses, offering renewed hope for patients with treatment-refractory malignancies.

### 4.2 Enhancing T Cell Function via TGF-β Payload and immune checkpoint blockade

TGF-β is a key regulator of immune homeostasis, but in cancer, it fosters an immunosuppressive TME by inhibiting T cell activation, proliferation, and cytotoxicity. This allows tumors to evade immune surveillance. Blocking TGF-β signaling within tumors is a promising strategy to restore T cell function and enhance cancer immunotherapy. OVs engineered to deliver TGF-β inhibitors offer a localized and potent approach to achieve this, converting immune-excluded tumors into highly inflamed tumors that are more responsive to immunotherapy (121).

Genetically modified OVs can express TGF-β inhibitors, including small molecules, decoy receptors, or neutralizing antibodies, ensuring targeted blockade of TGF-β signaling within the TME while minimizing systemic toxicity. TGF-β signaling suppresses CTLs and enhances the function of Tregs and MDSCs, all of which contribute to immune evasion (122). By disrupting this pathway, OVs enhance T cell metabolism, cytokine production, and effector function, while reducing immunosuppressive cell populations and promoting sustained antitumor immunity (123).

Several OV platforms incorporating TGF-β blockade are under investigation. Seneca Valley virus (SVV-001) induces tumor lysis and reprograms the immune microenvironment to enhance T cell infiltration. AdAPT-001, an oncolytic adenovirus (oAdV) expressing a TGF-β trap, has shown promising results in a Phase 1 trial, particularly when combined with ICIs, yielding durable responses in refractory tumors such as sarcomas and triple-negative breast cancer (124, 125). Jurona virus (JURV), in combination with anti-PD-1 therapy, improved survival and immune activation in hepatocellular carcinoma models, with favorable tolerability (14).

VacV engineered to express TGF-β receptor (TGF-βR) inhibitors represents another potent approach. By delivering these inhibitors directly into tumors, VacV suppresses TGF-β-mediated immune evasion, enhances T cell recruitment and cytotoxicity, and significantly improves ICI efficacy in preclinical models (126, 127).

Although early findings are encouraging, further research is needed to optimize delivery, specificity, and immune modulation. Ongoing clinical trials will be critical in determining the safety and efficacy of TGF-β-blocking OVs. Their ability to reshape the TME, synergize with ICIs (e.g., anti-PD-1 and anti-CTLA-4 therapies), and directly enhance T cell function positions them as powerful tools in the next generation of cancer immunotherapy (40, 128). While checkpoint blockade alleviates T cell exhaustion, OVs enhance T cell metabolic fitness and functional capacity, overcoming multiple layers of tumor-mediated immune suppression. This combination strategy has demonstrated enhanced tumor regression and prolonged survival in preclinical models, underscoring its potential for clinical translation (124, 125). In a Phase 1 clinical trial, AdAPT-001 exhibited encouraging responses, particularly when combined with ICIs, yielding a high objective response rate and durable clinical benefit in patients with refractory tumors, such as sarcomas and triple-negative breast cancer (124, 125). Jurona virus (JURV), a novel OV, in combination with anti-PD-1 antibody, enhanced immune response and improved survival in HCC models (14). The therapy demonstrated favorable tolerability, with most adverse events being mild and transient, further supporting its clinical potential.

### 4.3 Overcoming physical barriers in TME

#### 4.3.1 Enhancing TME and cytoskeleton remodeling for OV penetration

The TME serves as a physical barrier that restricts viral diffusion and immune cell infiltration. To overcome this, OVs have been engineered to express matrix metalloproteinases (MMPs) and other proteolytic enzymes that degrade ECM components, facilitating deeper tumor penetration (129). For example, recombinant measles viruses expressing MMP-activated fusion proteins selectively target MMP-rich tumors, enhancing both safety and oncolytic activity (130, 131). Similarly, AdVs and VacVs modified to express MMPs or urokinase-type plasminogen activator (uPA) improved viral dissemination and tumor infiltration (102, 132). A recombinant oVacV encoding hyaluronidase (OVV-Hyal1) was shown to degrade hyaluronic acid, a major ECM component, which significantly improved the intratumoral spread of the virus and enhanced the delivery of co-administered therapies like doxorubicin, gemcitabine, and CAR T cells. The virus also promoted the infiltration of immune cells such as CD8+ T cells and NK cells, amplifying the antitumor immune response (133).

OVs hijack intracellular transport and induce skeletal remodeling. They exploit the host cell’s microtubule transport system to reach the nucleus, where replication often occurs. African swine fever virus uses its p54 protein to bind to dynein light chain, DYNLL1, hijacking the dynein motor complex (134). Other OVs, such as oAdVs and oHSV similarly use dynein-mediated transport to move from the cell membrane to the nucleus. Some viruses have evolved proteases that cleave a dynein-activating adaptor protein, Ninein-like, disabling its function and making the tumor cells more permissive to OV infection (135).

In parallel, OVs can disrupt the cytoskeleton architecture of tumor cells by modulating signaling pathways such as Rho/ROCK and PI3K/Akt/mTOR, which regulate cytoskeletal dynamics. This disruption leads cytopathic effects such as cell rounding, detachment, and death, weakening the tumor’s structural integrity and promoting ICD. OVs like reovirus and AdVs can disrupt actin stress fibers and microtubule networks in infected tumor cells, enhancing viral spread and immune activation (55).

#### 4.3.2 Hypoxia-responsive viral engineering

Hypoxia-responsive viral engineering is a promising strategy in OVT that exploits the oxygen deprivation in the TME to enhance selectivity and safety of OVs. Hypoxia, a hallmark of the TME, arises from rapid proliferation of tumor and inadequate vascularization, and it often impairs cell infiltration and reduces the efficacy of conventional therapies. However, this hostile environment can be leveraged to improve the precision of OV-based treatment. To exploit this feature, hypoxia-responsive elements (HREs), which are activated by hypoxia-inducible factors (e.g. HIF-1α), have been incorporated into essential viral genomes to regulate essential replication genes such as E1A in AdVs (130). These modifications enable OVs to replicate preferentially in hypoxic tumor regions, where immune defenses are often suppressed. In normoxic tissues, HIF-1α is rapidly degraded, keeping HRE-controlled genes inactive and thereby minimizing off-target viral replication. In contrast, in hypoxic tumor zones, HIF-1α binds to HREs and initiates the transcription of viral genes, enabling selective replication and oncolysis within the tumor. A well-characterized example of this approach involves AdVs engineered with the E1A gene under HRE control, allowing replication specifically in hypoxic tumor cells. Additionally, viruses like VSV naturally thrive in such environments, making them ideal candidates for hypoxia-adapted OVT (29). Beyond replication control, some OVs are designed to express therapeutic payloads, such as cytokines or prodrug-converting enzymes, only under hypoxic conditions, further enhancing tumor specificity and therapeutic impact.

Moreover, the integration of hypoxia-responsive elements with other engineering strategies, such as the expression of ECM-degrading enzymes, enables OVs to overcome both structural and molecular barriers within the TME. These combined modifications not only improve viral dissemination and tumor selectivity but also synergize with immunotherapies, offering a multifaceted approach to cancer treatment. Overall, hypoxia-responsive viral engineering represents a powerful tool in the development of next-generation OVs capable of targeting resistant tumor regions with high precision.

### 4.4 Enhancing OVT through physical modalities

To overcome challenges of OVT as monotherapy, such as tumor heterogeneity, immune evasion, and the immunosuppressive TME as discussed in section 2, combination strategies have emerged as a powerful means to potentiate OVT. The integration of OVs with ICIs, chemotherapy, radiotherapy, or targeted agents, has amplified antitumor responses, improved viral delivery, and sustained immune activation, thus enhancing therapeutic outcomes of OVT across diverse cancer types. Combination of Tumor Treating Fields (TTFields) and radiation therapy with OVT offers an innovative therapeutic synergy to improve tumor targeting, viral spread, and immune engagement.

#### 4.4.1 Tumor treating fields

TTFields represent a non-invasive treatment modality that delivers low-intensity, intermediate-frequency alternating electric fields to disrupt mitosis by interfering with microtubule polymerization, thereby inducing cell cycle arrest and (136). Clinically TTFields have demonstrated efficacy in GBM, malignant pleural mesothelioma, and mesothelioma, predominantly affecting actively dividing cells. When combined with OVs, which selectively infect both dividing and non-dividing tumor cells, this dual-targeting approach expands the therapeutic window and enhances cancer cell susceptibility. In addition to their direct antitumor effects, TTFields can modulate the TME by improving vascular perfusion, thereby facilitating viral dispersion and enhancing tumor tissue penetration. These effects, when coupled with the immunogenic potential of OVs, which promote the release of TAAs and danger signals (DAMPs), may synergistically amplify immune cell recruitment and foster durable antitumor immunity (136, 137). Nevertheless, further research is warranted to optimize treatment protocols, identify predictive biomarkers, and elucidate the precise molecular mechanisms underlying the synergistic interactions between TTFields and OVs (138, 139).

#### 4.4.2 Radiation therapy

Radiation therapy in combination with GL-ONC15 enhances OVT efficacy via DNA damage, ICD, and TME reprogramming (140, 141). The intersection of these mechanisms offers a powerful therapeutic synergy. The immune-stimulating effects of OVs augment radiation therapy (142). OVs facilitate antigen presentation and immune system activation when combined with radiation-induced immune priming, results in a more robust and sustained antitumor immune response (143) which triggered necroptotic cell death, releasing DAMPs and shifting the macrophage M1/M2 ratio, promoting a more pro-inflammatory and antitumor immune landscape (144). Radiation-induced cell stress and necrosis not only provide substrates for OV amplification but also upregulate viral entry receptors, rendering tumor cells more susceptible to infection. Furthermore, radiation primes the immune system by boosting antigen presentation and immune infiltration. Combined with the innate immune stimulation from OVs, this strategy significantly enhances antitumor responses. Preclinical models, the combination of high-dose hypo-fractionated stereotactic body radiotherapy (SBRT) and oncolytic VacV in glioma and sarcoma, demonstrate increased infiltration of activated T cells (CD4^+^, CD8^+^), reduced Tregs, and improved survival outcomes (145). The combination of Delta-24-RGD and radiotherapy in pediatric gliomas and DIPG led to impaired DNA damage repair, increased immune cell trafficking, and prolonged survival (145). As ongoing trials continue to refine these combinations, this multimodal strategy represents a promising frontier for durable and effective cancer immunotherapy.

### 4.5 Enhancing OVT with inhibitors of aberrant signaling pathways

#### 4.5.1 Combination with antagonist of PI3K/Akt/mTOR pathway

The PI3K/AKT/mTOR signaling pathway plays a central role in cellular growth, proliferation, and survival, making its dysregulation a hallmark of various cancers and a critical target for therapeutic intervention (146–148). Phosphoinositide 3-kinase/protein kinase B/mechanistic target of rapamycin (PI3K/Akt/mTOR) inhibitors combined with OVs offers a promising strategy to enhance tumor susceptibility to oncolysis and modulate TME for better outcomes. Combination of OV ZD55-TRAIL with LY294002, a PI3K inhibitor, in multiple myeloma, induces apoptosis via caspase activation, inhibits IGF-1R and NFκB and enhanced oncolysis (149). Also, combination of ZD55-TRAIL with MG132, a proteasome inhibitor, increased death receptor 5 expression, and enhanced oncolysis only in cancer cells (150).

The VC2, a novel oHSV-1, promoted long-lasting systemic anti-melanoma immune responses and improved survival in an immunocompetent B16F10-derived mouse melanoma model (151). Also, combination of reovirus and rapamycin enhanced viral oncolysis by inhibiting mTOR activity in B16F10 melanoma cells (152). Although rapamycin did not affect reovirus-neutralizing antibodies or cell cycle effects, it reduced viral replication and reovirus-induced apoptosis (152). Conversely, rapamycin enhanced autophagy, increased adenoviral E1A expression, and improved the replication of Ad-cycE, an oAdV, enhancing oncolysis than monotherapy (153).

Resistance to PI3K/AKT inhibitors often results from hyperactivation of Wnt/β-catenin signaling. Inhibition of tankyrase 1, regulates Wnt/β-catenin promotes replication of β- and γ-variants while suppressing wildtype of HSV-1, and offers promising OVT which warrants further exploration (154). Combination of HSVs with PI3K/AKT inhibitors, effectively target GBM and prostate cancer stem-like cells (139). AdV (150) and Newcastle Disease Virus (155, 156) in combination with inhibitors of the PI3K/AKT/mTOR pathway also enhanced oncolysis. However, temozolomide, gold standard of GBM treatment which activates Wnt/β-catenin signaling through the PI3K/AKT pathway (157), potentially counteract OVT in combination with G47Δ-mIL12 (an HSV-1 variant) (158). Combining Ovs with PI3K inhibition remodels the TME, and restores ICI sensitivity (159).

PI3Kδ inhibition enhances systemic OV delivery. IC87114 or idelalisib, clinically approved PI3Kδ inhibitor, improved the oncolysis of intravenously administered VacV by preventing sequestration of systemic macrophages, disrupting RhoA/ROCK, AKT, and Ras signaling, and promoting viral spread, in pancreatic cancer (160).

mTOR is a key regulator of cell proliferation and survival, making it a promising target for combination therapies with OVs (161). Everolimus (RAD001), an mTOR inhibitor, in combination with AdVs enhances oncolysis in colon cancer, inhibits tumor growth, mitigates angiogenesis, and suppresses immune responses (162). Combination of rapamycin with myxoma virus, VacV, HSV, VSVΔM51, and AdV enhances oncolysis and viral replication only in cancer cells by disrupting mTORC1-dependent type I IFN production and reducing macrophage infiltration in tumors (152, 163, 164). Interestingly, inhibitors targeting both mTORC1 and mTORC2, unlike rapamycin, enhance HSV1-dICP0 infection through the eIF4E/4E-BP pathway, further highlighting the potential of PI3K/AKT/mTOR inhibition in OVT (164).

In summary, combining of OV with PI3K/AKT/mTOR pathway inhibitors, enhances viral replication, and reshapes the immune milieu. Further preclinical and clinical investigations are needed to optimize treatment protocols, identify predictive biomarkers, and address resistance mechanisms.

#### 4.5.2 Combination with JAK/STAT pathway modulators

The JAK/STAT pathway is a key signaling cascade involved in cellular responses to cytokines and growth factors. Upon ligand binding, intracellular JAKs phosphorylate and activate STAT proteins that regulate gene expression (165). Hyperactivation of this pathway increases Tregs gene expression, fostering tumor tolerance and immune suppression (165). Tumors with defective IFN-β signaling are more susceptible to OV-mediated oncolysis, while those with intact IFN pathways mount antiviral response that hinders OV replication and efficacy (32, 166). Hence, JAK/STAT inhibitors have emerged as promising adjuncts to OVT (167). Ruxolitinib, a selective JAK1/2 inhibitor, enhances replication and efficacy of VSV-IFN-β and VSV-ΔM51 in non-small cell lung cancer (NSCLC) and human PDACs, respectively, by inhibiting STAT1/2 phosphorylation and reducing antiviral responses that restrict viral replication (167, 168). Similarly, TPCA-1, a dual inhibitor of JAK1 and IκB kinase, boost HSV replication in malignant peripheral nerve sheath tumor (MPNST) cells, amplifying OV-mediated cytotoxicity (169).

Beyond the JAK/STAT axis, RNA viruses and poxviruses encounter additional barriers such as protein kinase R (PKR), which activates stress responses via the NF-κB and c-Jun N-terminal kinase (JNK) pathways (170). JNK’s role in viral replication is context-dependent; its inhibition can enhance viral replication in some cases. For instance, combining the JNK inhibitor SP600125 with VacV significantly increased viral titers and induced apoptosis in murine fibroblast cells (171). Further research is needed to optimize these and improve their outcomes in oncology.

#### 4.5.3 Targeting p53 and MDM2 pathways to sensitize tumors to oncolytic viruses

The tumor suppressor, p53 protein, maintains genomic stability by inducing cell cycle arrest, DNA repair, senescence, and apoptosis in response to cellular stress (172). Its mutation found in about 50% of cancers subtypes, contribute to tumor growth and progression (173). ONYX-015, an oAdV, combined with cisplatin and 5-fluorouracil enhances oncolysis in p53-deficient cancer cells, particularly recurrent head and neck tumors (174).

Also, targeting MDM2-p53 interaction restore p53 function in tumors with wild-type p53, making them more susceptible to OVT (175–177). MDM2, an E3 ubiquitin ligase, negatively regulates p53 by promoting its degradation. Combining an MDM2 inhibitor with Ad-delE1B, an AdV lacking the E1B-55kDa gene, enhanced viral replication and oncolysis in mesothelioma cells with wild-type p53. This approach increased nuclear factor 1 expression and amplified oncolysis (175) offering a promising strategy for treating p53-mutant or -deficient cancers by restoring p53’s tumor-suppressive functions.

#### 4.5.4 Combination with inhibitors of EGFR/K-RAS/MAPK signaling pathway

Receptor tyrosine kinases (RTKs) are essential enzymes involved in cellular processes such as growth, motility, differentiation, inflammation, and metabolism. Their dysregulation is a hallmark of many cancers, making them attractive therapeutic targets (178). Combining oncolytic viruses (OVs) with RTK inhibitors offers a synergistic strategy to enhance tumor selectivity, viral replication, and immune activation.

EGFR-targeted therapies have shown promise in combination with OVs. For instance, erlotinib combined with canerpaturev (C-REV) enhances viral replication and reduces tumor burden in colorectal cancer models. Similarly, cetuximab, an EGFR inhibitor, improves C-REV distribution and inhibits angiogenesis, leading to tumor regression in HT-29 xenografts (179). In EGFR-driven tumors such as malignant peripheral nerve sheath tumors (MPNSTs), combining oncolytic HSV (oHSV) with erlotinib enhances viral biodistribution and therapeutic efficacy (79).

Multi-target RTK inhibitors like sorafenib and sunitinib, which block VEGF, PDGF, and ERK pathways, have demonstrated synergistic effects with OVs. Sorafenib, approved for advanced renal cell carcinoma (RCC) and hepatocellular carcinoma (HCC), enhances OV efficacy by sensitizing tumor vasculature and inhibiting angiogenesis (180, 181). When combined with VSV, sunitinib suppresses antiviral enzymes (PKR, RNase L), boosting viral replication and tumor clearance in prostate, breast, and kidney cancer models. Similar synergy has been observed with VacV and reovirus in pancreatic neuroendocrine tumors and RCC (182, 183).

A notable example is the sequential use of Pexa-Vec (JX-594) followed by sorafenib in HCC, where Pexa-Vec primes the tumor for VEGF/VEGFR inhibition, enhancing sorafenib efficacy (184, 185). Likewise, combining axitinib (a VEGFR inhibitor) with G47Δ-mIL12 improves antitumor responses in glioblastoma models. CSF-1R inhibitors like PLX3397 also synergize with OVs by reprogramming the TME, increasing CD8^+^ T cell infiltration, and enhancing anti-PD-1 therapy (43, 186).

In RAS-mutated tumors, combining OVs with RAS/BRAF/MEK inhibitors enhances viral replication and overcomes resistance. Examples include combinations of reovirus (TR3D) and PLX4720, T-VEC and trametinib, and VacV and trametinib, which have shown improved outcomes in melanoma, ovarian cancer, and BRAF-mutant tumors (187–190).

Together, these combination strategies not only suppress tumor growth but also overcome resistance mechanisms, enhance immune responses, and improve viral delivery. The growing body of evidence supports RTK inhibitor, OV combinations as a powerful approach for treating aggressive and treatment-resistant cancers.

### 4.6 Combination with drugs, nutraceuticals, and their synthetic analogs

The combination of OVs with drugs, nutraceuticals, and their synthetic analogs, has emerged as a promising strategy in cancer therapy. Over the past decade, these small molecules have been explored for their ability to modulate key signaling pathways, enhance immune responses, and reshape the immunosuppressive TME (191). Their potential to augment OVT has opened new avenues for combination therapies, offering enhanced therapeutic efficacy and improved patient outcomes. They work synergistically with OVs by complementing and enhancing OV-mediated tumor destruction (192). They can modulate intracellular signaling, increase viral replication, improve tumor selectivity, and promote ICD, thereby maximizing the antitumor effects of OVs. Immune-modulating agents can also counteract the immunosuppressive TME, making it more susceptible to OV infection and immune activation. Additionally, these agents may sensitize tumor cells to OV-induced apoptosis or inhibit pathways that allow cancer cells to evade viral oncolysis.

The structure of anticancer drugs directly contributes to their capacity to enhance OVT by targeting complementary pathways in cancer cells and the TME (**Table 1**). HDAC inhibitors such as valproic acid and trichostatin A use their zinc-chelating functional groups to disrupt chromatin condensation and restore expression of viral entry receptors and immunostimulatory genes, sensitizing tumors to viral infection and immune-mediated clearance (193, 194). Multi-kinase inhibitors like sunitinib, whose indolinone scaffold allows ATP-site binding across receptor tyrosine kinases, suppress angiogenesis and impair antiviral signaling pathways such as IFN responses, thereby promoting viral replication within tumors (182, 183, 195). Lenalidomide’s phthalimide core enables targeted degradation of transcription factors critical for tumor survival and immune evasion, indirectly boosting antiviral immunity and modulating the TME to favor OVT (196–199). Bortezomib’s boronic acid moiety blocks proteasomal degradation of pro-apoptotic proteins, increasing virus-induced cell death. Doxorubicin, through its planar anthracycline core intercalation and free radical generation, compromises DNA integrity and heightens susceptibility to viral oncolysis (200). Everolimus leverages its macrocyclic lactone structure to inhibit mTORC1, a pathway critical both for tumor growth and for type I interferon production, thereby enhancing viral replication and spread (201–203). Platinum-based drugs like cisplatin and carboplatin, with their reactive platinum coordinated to reactive ligands that form intra- and interstrand DNA crosslinks ultimately triggering apoptosis and synergizing with viral lytic activity (204–206). Taxanes and camptothecin derivatives such as paclitaxel, docetaxel, and irinotecan, possess complex diterpenoid cores that stabilize microtubules and prevent their depolymerization, arresting mitosis or topoisomerase function, sensitizing dividing cancer cells to virus-induced apoptosis. Antimetabolites like 5-fluorouracil and gemcitabine, by depleting nucleotide pools and incorporating into nucleic acids, create replication stress that increases tumor permissiveness to viral infection (207, 208). Collectively, the unique structural features of these agents not only define their primary anticancer mechanisms but also strategically prime tumors to respond more robustly to oncolytic viruses, offering a rational basis for combination therapies that exploit vulnerabilities in cancer cell survival, antiviral defense, and immune regulation.

**Table 1:** Combination of OV with drugs.

Combination therapy with OVs	Tumor type	Structure of small molecules	Targets and structure-activity relationship of drugs in enhancing OVT	References
T-VEC + Cyclophosphamide or Paclitaxel	Nonmetastatic TNBC	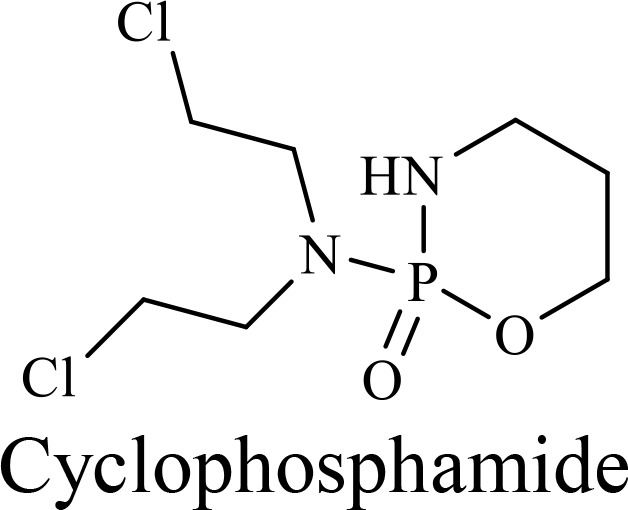 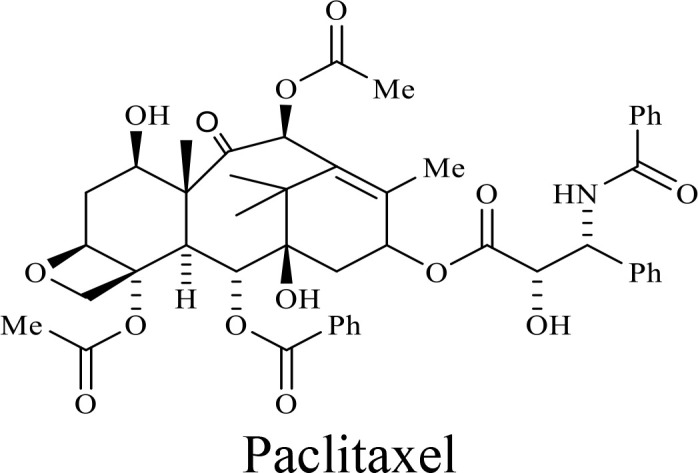	Taxanes and camptothecin derivatives such as paclitaxel, docetaxel, and irinotecan, have pentacyclic ring that disrupt microtubule dynamics or topoisomerase I function, sensitizing dividing cancer cells to virus-induced apoptosis. They arrest G2/M phase of the cell cycle and activate c-Jun N-terminal kinase (JNK) signaling pathway and disrupt topoisomerase-II-mediated DNA repair. Cyclophosphamide is metabolized into phosphoramide mustard, a cytotoxic, bifunctional alkylating agent. Its two reactive chloroethyl groups undergo intramolecular cyclization to form aziridinium ions, which alkylate DNA at the N7 position of guanine, resulting in interstrand and intrastrand cross-links. This DNA damage induces cell cycle arrest and apoptosis, particularly in rapidly dividing tumor cells. Additionally, phosphoramide mustard contributes to the depletion of proliferating immune cells, especially lymphocytes, leading to immunosuppression within the TME. This effect is critical in OVT, as it reduces antiviral antibody titers, especially when cyclophosphamide is administered systemically. By dampening immune clearance, it enhances viral persistence and tumor targeting.	(204, 259)
Rhabdovirus Maraba-MG1 + Paclitaxel	Breast cancer			(204, 260)
Adenovirus and paclitaxel encapsulated in extracellular vesicles (EV)	Lung cancer		Paclitaxel causes mitotic arrest at G2/M phase by stabilizing microtubules and disrupting spindle dynamics, leading to mitotic catastrophe, followed by intrinsic apoptosis, primarily through mitochondrial pathways. These events aid viral spread and immune activation, modulating TME to favour OV persistence	(204, 261)
Recombinant oncolytic myxoma virus + Gemcitabine	TNBC	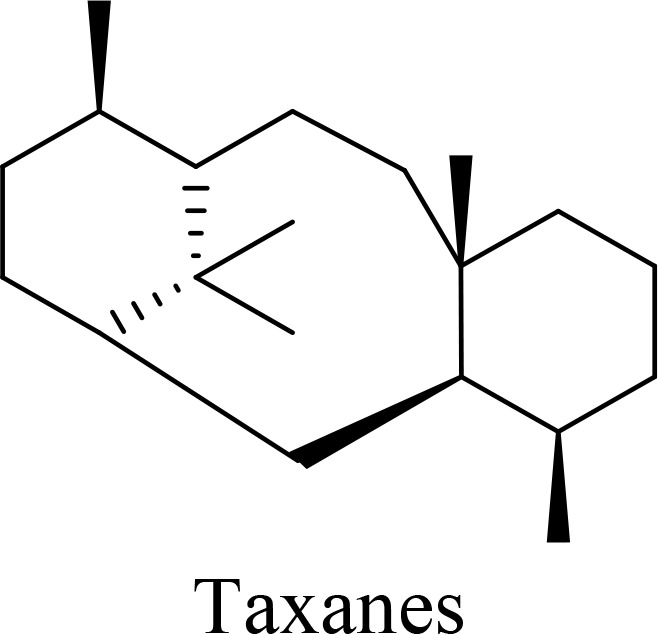 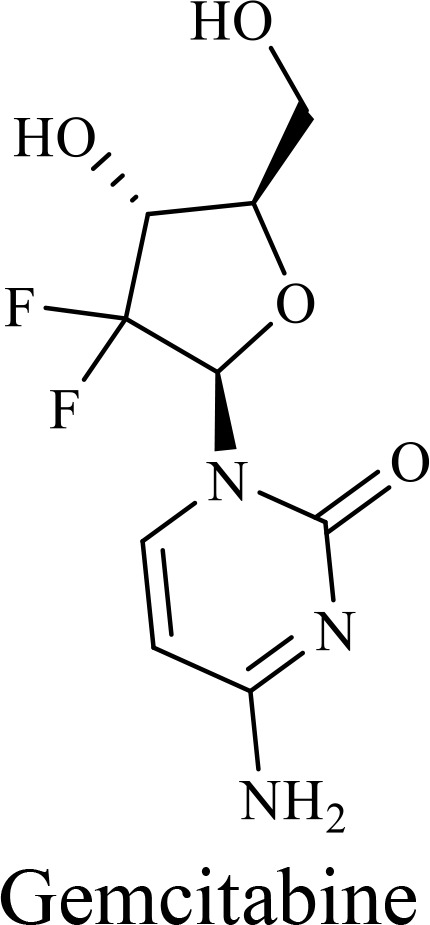	5-Fluorouracil (5-FU) and gemcitabine, a difluorinated nucleoside analog, exert their antitumor effects by inhibiting thymidylate synthase and ribonucleotide reductase, leading to nucleotide pool depletion. 5-FU is converted intracellularly into 5-fluoro-2'-deoxyuridine monophosphate (FdUMP) which forms a stable ternary complex with thymidylate synthase and 5,10-methylenetetrahydrofolate (a folate cofactor). This complex irreversibly inhibits thymidylate synthase, preventing the conversion of deoxyuridine monophosphate (dUMP) to deoxythymidine monophosphate (dTMP). This results in thymidine nucleotide depletion, leading to DNA synthesis arrest, replication stress, and cell death. Gemcitabine is phosphorylated to its active diphosphate (dFdCDP) and triphosphate (dFdCTP) forms. dFdCDP inhibits ribonucleotide reductase, reducing the pool of deoxynucleotides, including dTMP while dFdCTP is incorporated into DNA, causing chain termination. Its indirect depletion of nucleotide pools contributes to replication stress and DNA damage. The replication stress cause by these agents sensitizes tumor cells to OV infection by disrupting DNA repair and cell cycle progression, thereby enhancing viral replication and oncolysis.Gemcitabine also suppresses innate immune responses (NK cells, macrophages, and IFN-γ) and enhances OV persistence	(207, 208)
VacV + Gemcitabine	B16 tumors, Solid Tumors			(207, 262)
Myxoma Virus + Gemcitabine	Plasma cell neoplasms			(207, 263)
Adenovirus expressing relaxin (YDC002) + Gemcitabine				
HF10 (HSV-1 variant) + Gemcitabine or Taxanes	Myeloma			(207, 264)
VCN-01 + Gemcitabine, Nab-paclitaxel	Refractory multiple myeloma		Nab-paclitaxel (nanoparticle albumin-bound paclitaxel) which eliminates the need for toxic solvents (like Cremophor EL used in conventional paclitaxel), reducing systemic toxicity. It improves solubility and bioavailability of paclitaxel. Inhibit ribonucleotide reductase, mitotic arrest at G2/M phase	(207, 265)
Adenovirus + Rapamycin	Human breast cancer (MDA-MB-231), human lung cancer cell lines (A549 and H1299)	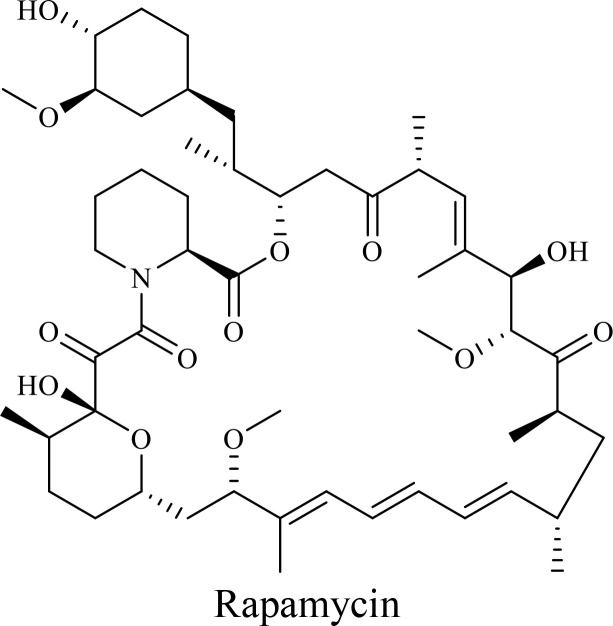	Rapamycin, a macrolide antibiotic with a large macrocyclic lactone ring, binds to the intracellular protein FKBP12 to form a complex that allosterically inhibits mTORC1. This inhibition suppresses tumor cell growth and angiogenesis while dampening type I IFN production and antiviral defense pathways that can limit OV replication. Structurally driven blockade of mTOR signaling therefore synergizes with OVT by enhancing viral replication, impairing tumor proliferation, and remodeling the tumor microenvironment to favor immune attack. It induces autophagy, enhances Virus protein expression and increases virus replication	(153, 266)
HSV-1 + Rapamycin	Resistant tumor cell line (MCF-7, MDA-MB-231, HeLa, HepG2 and HuH-7)			(153)
Reovirus + Rapamycin	Malignant melanoma			(152)
Pelareorep (Reolysin) + 5-fluorouracil, Gemcitabine, Irinotecan or Pembrolizumab	Pancreatic adenocarcinoma	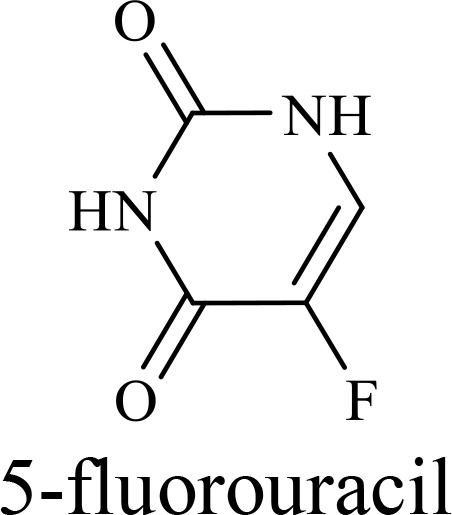 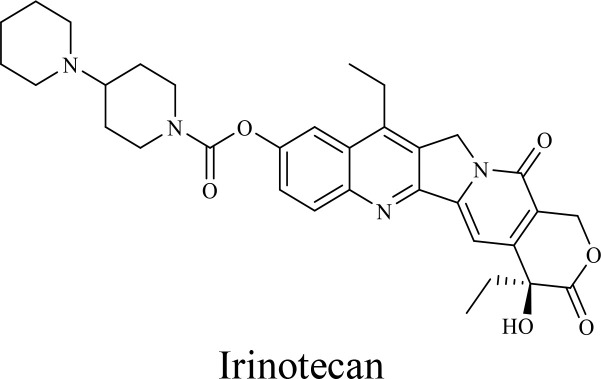 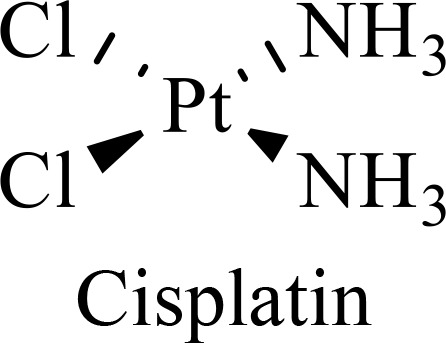	Cisplatin is a platinum-based compound with a square planar geometry. It contains two chloride ligands (leaving groups) and two amine groups (non-leaving groups). In the intracellular environment (low chloride concentration), the chloride ligands are replaced by water molecules, forming a reactive aquated complex that allows cisplatin to covalently bind to DNA, primarily at the N7 position of guanine and to form intrastrand and interstrand DNA cross-links, which distort the DNA helix and block replication and transcription. The structure of cisplatin enables it to form DNA cross-links that trigger cell cycle arrest, DNA damage, and immunogenic cell death. These effects weaken tumor defenses and enhance susceptibility to OVs, making cisplatin a valuable agent in chemo-virotherapy combinations. In addition, it blocks PD-1, inhibits ribonucleotide reductase. Cisplatin also induces cell death by mechanisms mediated by p53, p38 mitogen-activated protein kinase (p38 MAPKs) and/or the c-jun N-terminal kinases (JNK) activation, interference with the normal biosynthesis or function of nucleic acids	(207, 267)
ONYX-015+ Cisplatin + 5-fluorouracil	Recurrent head and neck cancer			(268)
GL-ONC1 (a VacV viariant) + Cisplatin and Radiotherapy	Locoregionally Advanced Head and Neck Carcinoma			(140, 268)
HSV-1 (NV1066) + Cisplatin	Malignant pleural mesothelioma			(268)
Myxoma Virus or Adenovirus subtype 5 or Adenovirus (LOAd703) + Paclitaxel + Cisplatin	Ovarian Cancer			(268–270)
adenovirus (Ad11 or Adenovirus D24‐RGD) + Cisplatin	Osteosarcoma			(268)
rMV-Hu191 + Cisplatin	Gastric cancer		Acid sphingomyelinase-mediated apoptosis	(268, 271)
Reovirus or Pelareorep + Docetaxel	Prostate cancer	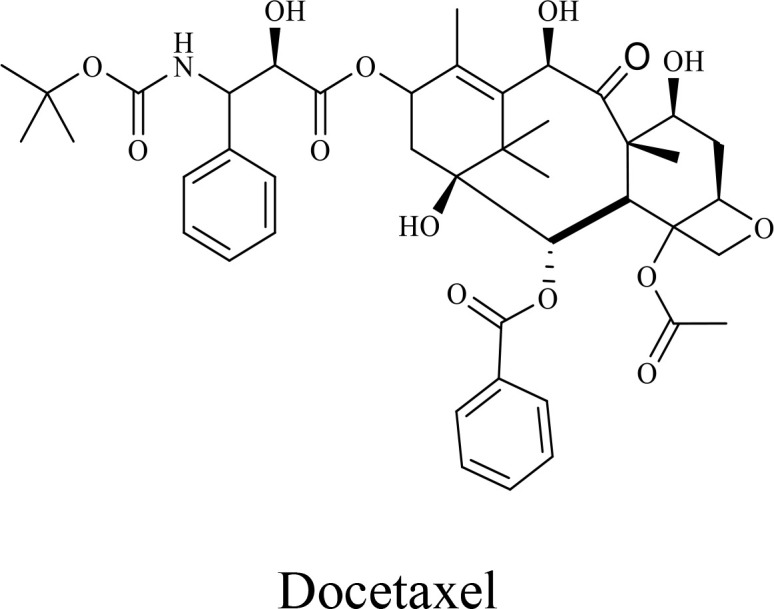	Inhibit microtubular depolymerization, attenuation of the effects of Bcl-2 and Bcl-xL gene expression.	(272, 273)
Adenovirus + Docetaxel	Castration-resistant prostate cancer		Docetaxel inhibits microtubular depolymerization, and attenuate the effects of Bcl-2 and Bcl-xL gene expression.	(273, 274)
Adenoviral agent OBP-401 + Docetaxel	Pancreatic cancer cell line (HaP-T1, PANC-1 and MIA Paca2)			(273)
Pelareorep+ Carboplatin and Paclitaxel	Metastatic or recurrent NSCLC	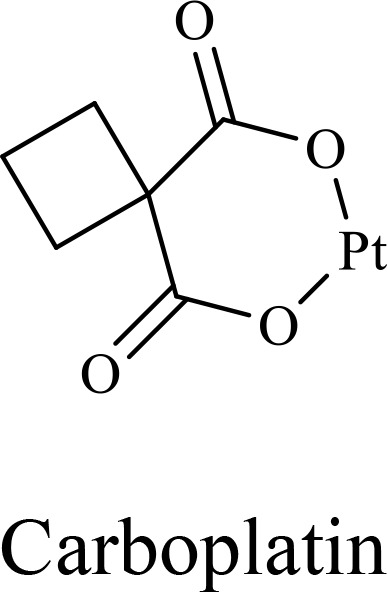	Carboplatin, with its reactive platinum centers, induce DNA crosslinks that synergize with viral lytic activity. Carboplatin interferes with DNA synthesis in the S phase of the cell cycle, targets microtubules	(204, 239)
Reovirus or Pelareorep + Carboplatin+ Paclitaxel	Metastatic pancreatic adenocarcinoma			(204–206)
Myxoma virus + Rapamycin	Glioma	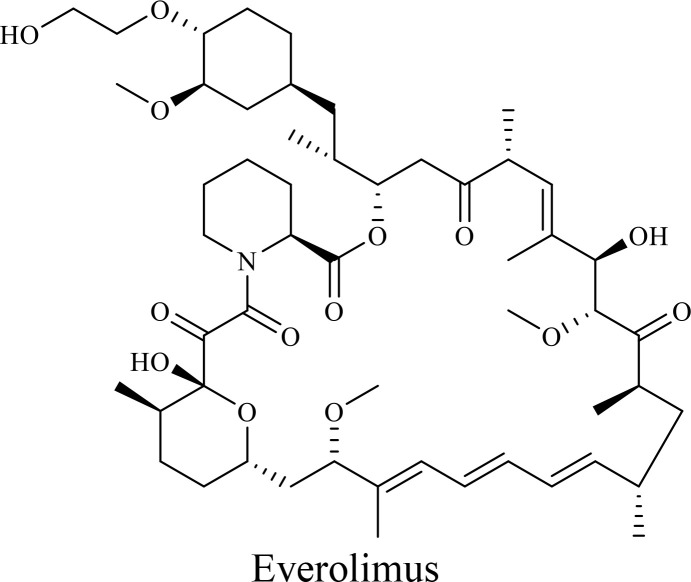	Everolimus a derivative of rapamycin, leverages its macrocyclic lactone structure to inhibit mTORC1, a pathway critical both for tumor growth and for type I IFN production, thereby enhancing viral replication and spread. binds FKBP12 to inhibit mTORC1, suppressing cellular proliferation and angiogenesis. While it modulates macrophage activity and cytokine release, it also induces autophagy, contributing to its antitumor effects. Hence its use in combination with OVT	(201–203)
Adenovirus Delta-24-RGD + Everolimus (RAD001)				
VSVΔ51-GFP + LCL161	Rhabdomyosarcoma	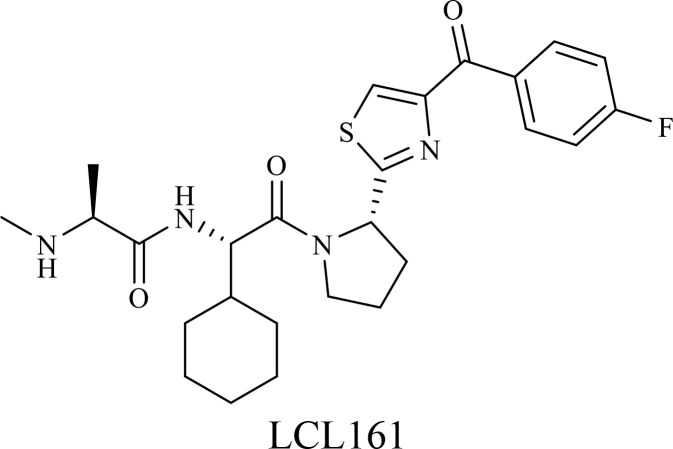	LCL161 is a small-molecule SMAC mimetic structurally designed to reproduce the N-terminal AVPI binding motif of endogenous SMAC. This peptidomimetic scaffold allows high-affinity binding to the baculoviral IAP repeat domains of inhibitor of apoptosis proteins such as cIAP1 and cIAP2. By antagonizing these proteins, LCL161 disrupts their interaction with caspases, enabling caspase activation and TNFα-mediated cell death. This mechanism sensitizes cancer cells to OV–induced apoptosis and amplifies immunogenic cell death, enhancing viral oncolysis and immune clearance	(275)
VacV + Doxorubicin	Ovarian Cancer	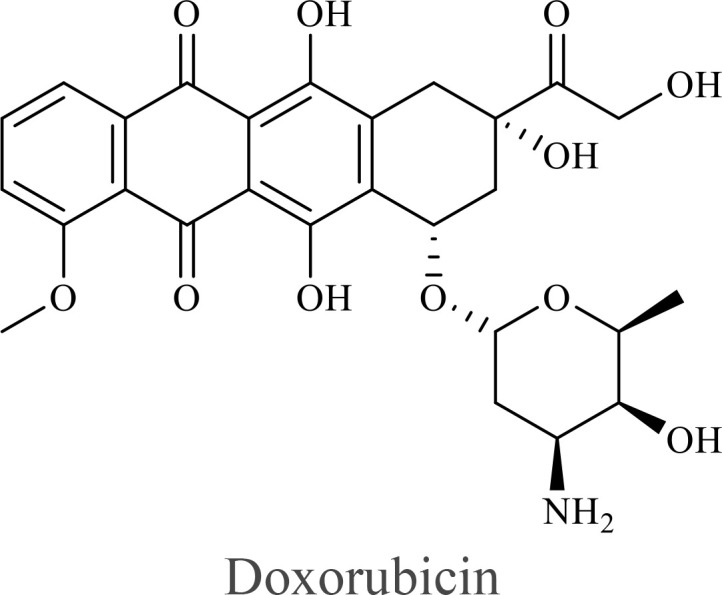	Doxorubicin, through its planar anthracycline core and an amino sugar moiety exerts its antitumor effects through DNA intercalation, free radical generation and stabilization of topoisomerase II–DNA complexes, compromising genomic integrity and promoting apoptosis. These mechanisms sensitize tumor cells to OV infection. In doxorubicin-resistant ovarian cancer, suppression of the MEK-ERK pathway enhances viral replication and cytotoxicity, supporting the therapeutic potential of combining doxorubicin with OVT.	(190, 200)
Reovirus + Carfilzomib + Perifisone	Multiple myeloma	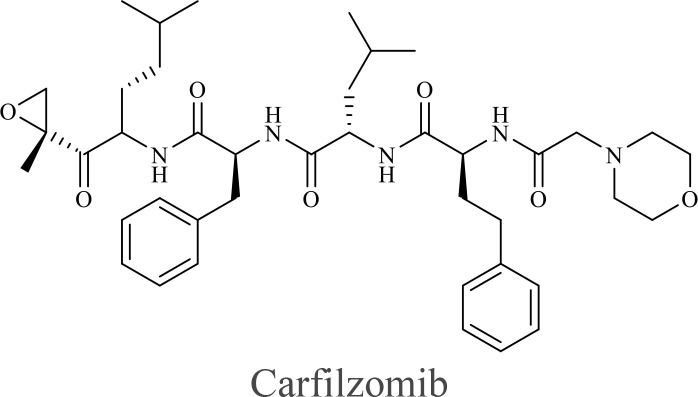	Carfilzomib, a tetrapeptide epoxyketone proteasome inhibitor, exerts its effects through an electrophilic warhead that forms an irreversible covalent bond with Akt and the N-terminal threonine of the proteasome’s catalytic β5 subunit. This stable morpholine adduct blocks proteolytic degradation of pro-apoptotic proteins and regulators of NF-κB signaling. As a result, carfilzomib not only drives tumor cell apoptosis but also impairs intrinsic antiviral responses that otherwise restrict oncolytic virus replication, creating a more permissive environment for viral propagation and tumor cell lysis.	(276, 277)
HSV or VSV + Bortezomib + PS-341	Adenocarcinoma A549 cells and myeloma cells	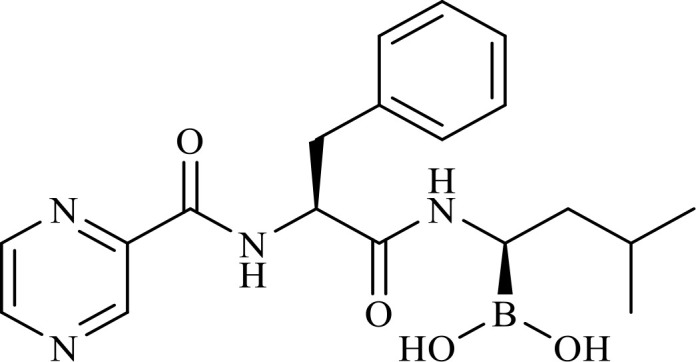	Bortezomib inhibits the 26S proteasome via its boronic acid moiety, leading to the accumulation of pro-apoptotic proteins and induction of apoptosis in cancer cells. The dipeptide boronic acid structure of bortezomib enables it to form a reversible covalent bond with the catalytic threonine of the proteasome. It enhances viral replication by inducing the unfolded protein response and upregulating HSP90, which supports viral polymerase activity. Bortezomib shifts virus-induced cell death from apoptosis to necroptosis, a more immunogenic form of cell death that can enhance antitumor immune responses. By sensitizing tumor cells to natural killer (NK) cell-mediated cytotoxicity, Bortezomib improves the efficacy of NK cell-based immunotherapies.	(240, 278–280)
Delta24-RGD + LBH589 and Scriptaid	Glioblastoma Cells	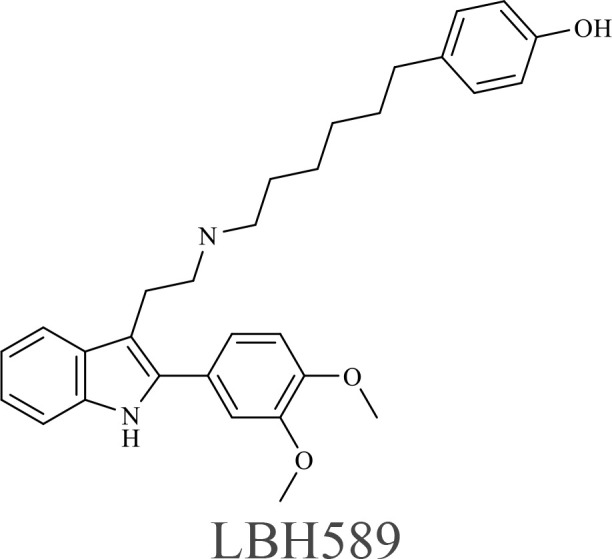 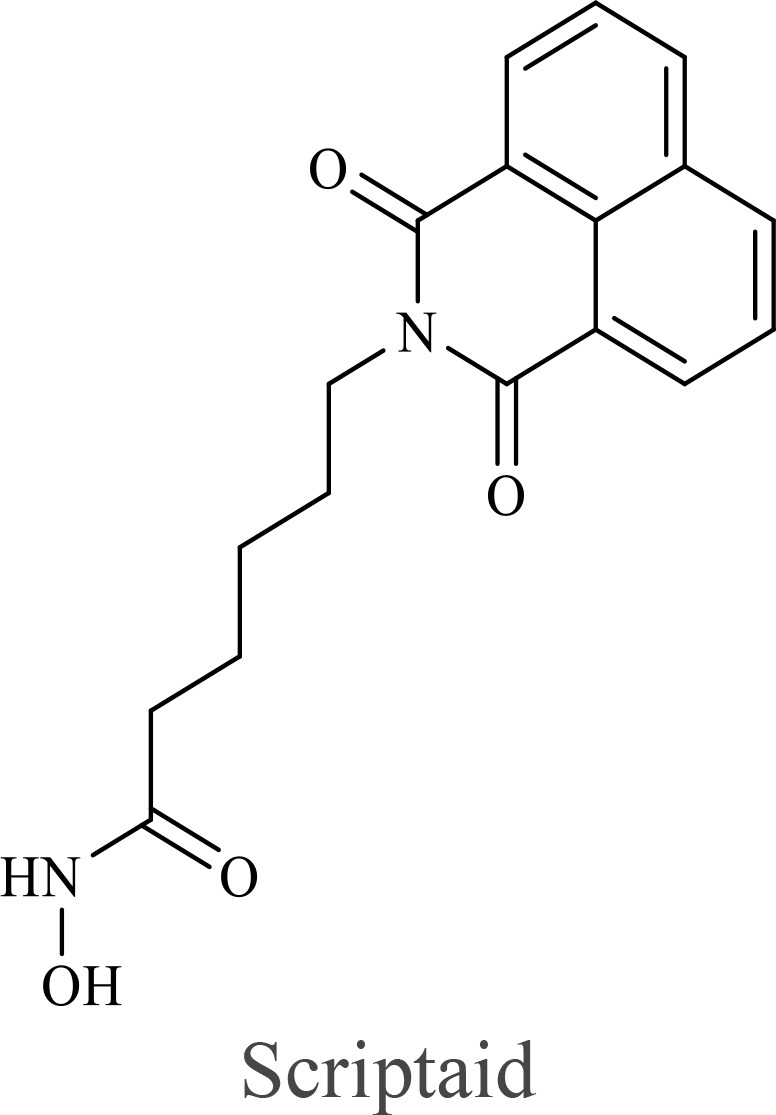	Scriptaid and LBH589 (panobinostat) are hydroxamic acid-based HDAC inhibitors that share a common structural principle: a zinc-chelating hydroxamate moiety linked by a flexible hydrophobic chain to an aromatic cap group. This configuration enables potent and broad-spectrum HDAC inhibition. By promoting histone hyperacetylation, these compounds relax chromatin structure, restore expression of silenced viral entry receptors (such as CAR and nectins), and upregulate immunostimulatory genes. Consequently, HDAC inhibitors not only facilitate viral infection and replication but also promote immune recognition of tumor cells following oncolytic virus–mediated lysis.	(281)
T-01 (HSV-1 variant) + Lenalidomide	Plasma cell neoplasms, multiple myeloma	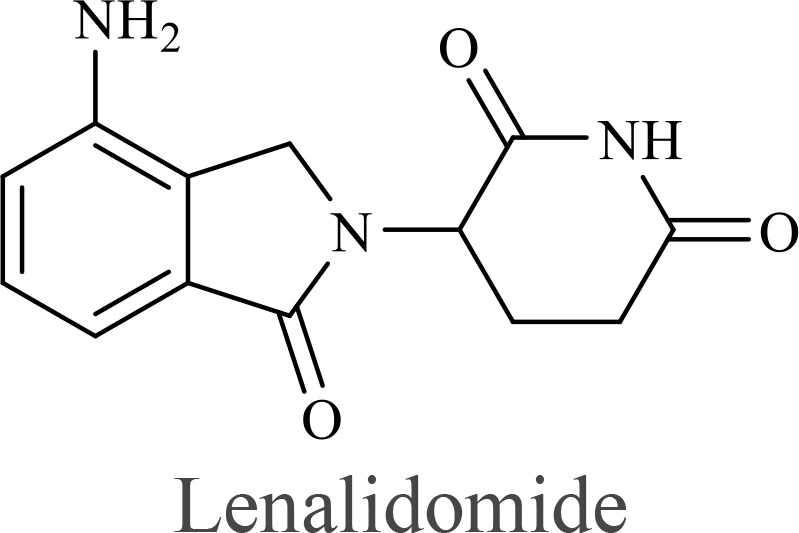	Lenalidomide a phthalimide derivative, binds the E3 ligase cereblon to promote degradation of transcription factors like IKZF1 and IKZF3, leading to anti-proliferative and immunomodulatory effects which are critical for tumor survival and immune evasion in multiple myeloma. This promotes tumor cell death and enhances immune responses by stimulating NK cell cytotoxicity and modulating the tumor microenvironment. These immunomodulatory effects support antiviral responses and have potential to synergize with OVT	(196–199)
VacV or Reovirus + Sunitinib	Pancreatic neuroendocrine tumors	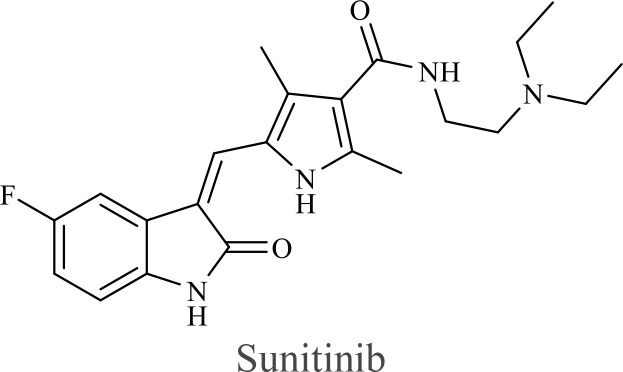	Sunitinib is a multi-kinase inhibitor with an indolinone-based planar aromatic core that targets receptor tyrosine kinases including VEGFR, PDGFR, and KIT by occupying their ATP-binding sites. This disrupts angiogenesis and tumor proliferation. In the context of oncolytic virotherapy, Sunitinib enhances viral distribution within tumors and suppresses antiviral innate immunity, thereby improving viral replication and therapeutic efficacy.	(182, 183, 195)
HSV + Trichostatin A (TSA)		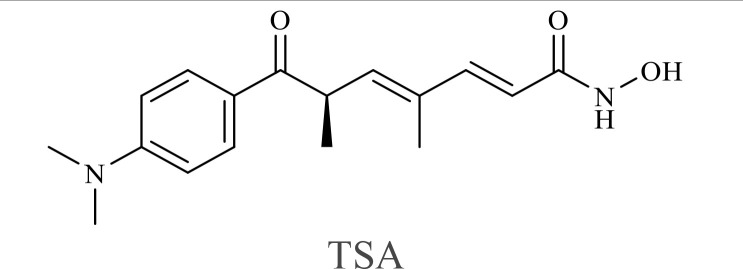	TSA, a hydroxamic acid-based HDAC inhibitor, chelates zinc in the HDAC active site and stabilizes binding through hydrophobic interactions. This inhibition leads to chromatin relaxation and reactivation of silenced genes, sensitizing tumor cells to OV infection. In combination with oHSV, TSA enhances antitumoral and antiangiogenic effects, promotes viral replication, and improves therapeutic efficacy.	(282–285)
HSV + Valproic Acid (VA)	Glioblastoma cells	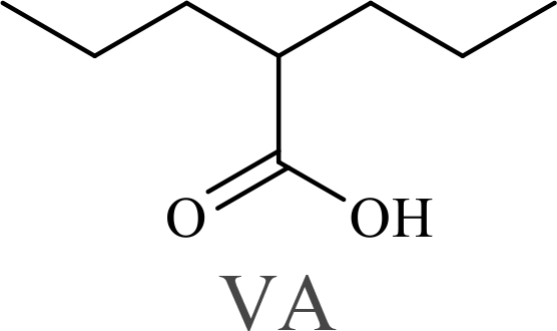	VPA is a histone deacetylase inhibitor whose carboxylic acid moiety contributes to zinc chelation within the HDAC catalytic pocket, leading to chromatin relaxation and reactivation of silenced tumor suppressor and immunomodulatory genes. This sensitizes tumors to OV infection. In combination with oHSV, VPA suppresses NK cell cytotoxicity by inhibiting STAT5 phosphorylation and T-BET expression, reducing IFN-γ production and enhancing viral persistence and antitumor efficacy.	(193, 194)

**Figure 3:**
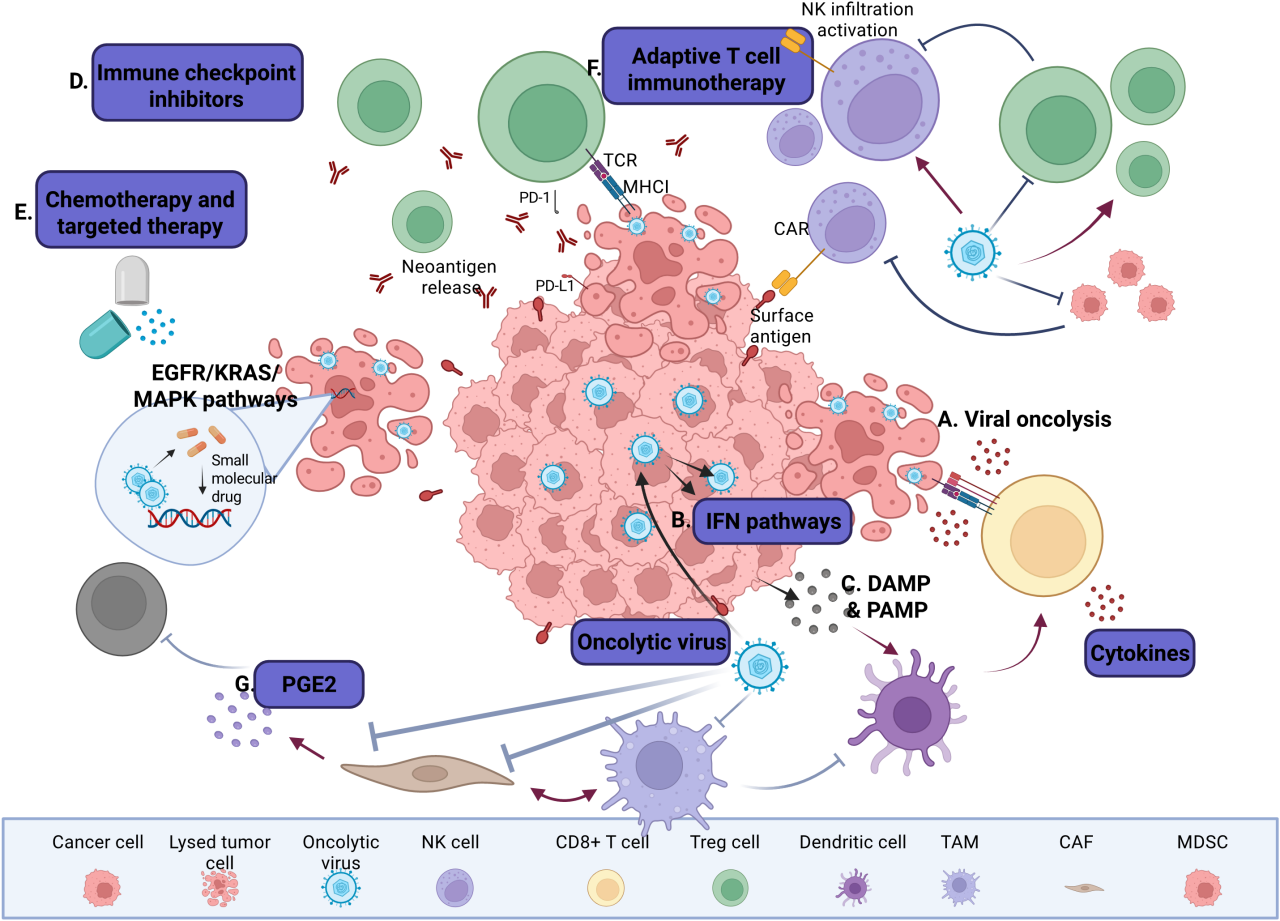
Interactions between OV and small molecules on tumors and TME. **(A)** OVs selectively replicate within tumor cells and mediate direct oncolysis. **(B)** Enhancement of OV-mediated release of DAMPs, PAMPs and proinflammatory cytokines promotes the infiltration of CTLs into the tumor beds and supports their cytolytic activity. **(C)** OVs activate IFN signaling pathways and stimulate innate and adaptive immune responses, leading to broader and more durable antitumor immunity. D) OV infection increases the expression of immune checkpoint molecules such as PD-L1 and CTLA-4 on tumor and stromal cells, rendering tumors more susceptible to ICIs. **(E)** Cytotoxic chemotherapies promote tumor cell death, often via ICD, while targeted therapies disrupt oncogenic signaling pathways and potentially tumor cell death. These treatments can elicit weak to moderate antitumor immune responses. **(F)** Relevant cells in the TME, such as TAMs, DCs, CAFs, and MDSCs, secrete ECM components, growth factors, and immunomodulatory cytokines, which can contribute to the regulation of tumor progression and therapeutic response in unique ways. For instance, CAFs suppress T and NK cell activity through secretion of factors such as PGE2 and TGF-β. Some OVs are engineered to target not only cancer cells, but also stromal cells and components such as CAFs. **(G)** OVs can reshape the TME by converting immunologically “cold” tumors into “hot” tumors, thereby enhancing immune cell recruitment and effector function (288).

Recent studies have identified several successful OV-small molecule combinations, highlighting both therapeutic potential and challenges (209). As cancer immunotherapy evolves, the strategic development of these small molecule as next-generation immunotherapeutic may redefine cancer treatment paradigms (210). The synergy between OVs and pharmacological small molecules can be categorized into distinct mechanisms that enhance tumor targeting and immune activation (**Figure 3**). However, further research and clinical trials are essential to optimize dosing regimens, identify predictive biomarkers, and address potential resistance mechanisms, ensuring this combinatorial strategy achieves its full therapeutic potential in oncology.

#### 4.6.1 Combination with epigenetic modulators

Histone deacetylases (HDACs) regulate transcription regulation and immune responses, particularly in IFN signaling (211). HDAC inhibitors are emerging as enhancers of OV replication in tumor cells, providing a novel therapeutic avenue. Among them, HDAC6 plays a crucial role in regulating immune responses and autophagy, especially in TLR-mediated signaling by intracellular bacteria (212). Targeting HDACs, can significantly improve OVs efficacy. For example, combining oncolytic G47Δ with the HDAC inhibitor trichostatin A (TSA) showed strong synergy against endothelial and various cancer cell lines, but not normal cells. This synergy depends on viral replication and high cyclin D1 levels in tumor cells, amplifying inhibition of cyclin D1 levels in tumour and VEGF to enhance treatment efficacy (212). In HCT116 colon tumor xenograft models, combining TSA and VacV improved survival compared to using either agent alone (79).

Valproic acid (VPA), an FDA-approved antiepileptic with HDAC-inhibitory properties has shown independent anticancer effects (213). With its carboxylic acid moiety to chelate the zinc ion within the HDAC catalytic pocket, thereby reactivating silenced tumor suppressor genes. In GBM models, VPA reduced NK cell and macrophage recruitment shortly after HSV infection, although infiltration increased over time. VPA also suppressed NK cell cytotoxicity *in vitro*. Conversely, combining VPA with H-1Protoparvovirus synergistically enhanced cytotoxicity and NS1-mediated transcription and cytotoxicity. This combination induced oxidative stress and increased apoptosis, leading to better therapeutic outcomes (214). Silent Mating Type Information Regulation 2 Homolog 1 (SIRT1), a NAD^+^-dependent histone deacetylase, has been linked to increased cancer cell susceptibility to OVT. For example, SIRT1 enhanced replication and oncolytic efficacy of VSVΔM51 in prostate cancer cells (PCC) (215). HDAC inhibitors like suberoylanilide hydroxamic acid (vorinostat), and resminostat upregulate miR-34a, which regulates SIRT1 levels, enhancing OV efficacy. Inhibiting or silencing SIRT1 also sensitizes prostate cancer cells to VSVΔM51, promoting viral replication and spread (215). The hydroxamic acid group of vorinostat is essential for its function, as it coordinates the zinc ion located in the catalytic site of HDAC enzymes, effectively inhibiting deacetylase activity while the suberoyl linker, an eight-carbon chain, allows the molecule to reach deep into the enzyme’s tubular pocket and position the zinc-binding moiety precisely. The terminal phenyl ring of the anilide functions as a capping group that sits at the rim of the active site, where it contributes important hydrophobic and π–π stacking interactions that stabilize binding. Resminostat shares the same fundamental pharmacophore with reminostat, a combination of a zinc-binding hydroxamic acid, a linker region, and an aromatic cap, but differs in key structural refinements that enhance its pharmacologic profile.

## 5 Clinical applications of OVs and vaccines

Clinical trials have extensively evaluated various OVs, both as monotherapies and in combination with ICIs and other treatments, demonstrating their significant potential in oncology (216, 217). Among the most well-characterized OVs, T-VEC has demonstrated remarkable efficacy in melanoma. As the only FDA-approved OV for melanoma, T-VEC has also approved in Australia and Europe for treating unresectable stages IIIB, IIIC, and IV melanoma (217, 218). The pivotal OPTiM Phase III trial revealed a durable response rate (DRR) of 25.2% for T-VEC-treated patients versus 1.2% for GM-CSF alone, with a median overall survival of 41.1 months versus 21.5 months in the control group (219, 220). Retrospective analyses in European cohorts reported an objective response rate of 63.7% and complete remission in 43.2% of treated patients (217).

Combinatorial strategies with T-VEC have also shown promise. A Phase II trial combining T-VEC with the anti-CTLA4 monoclonal antibody ipilimumab improved durable response rate to 29.6%, surpassing ipilimumab monotherapy (13%) (221, 222). Additionally, T-VEC combined with pembrolizumab, an anti-PD-1 drug, showed a twofold increase in response rates compared to T-VEC monotherapy (219, 223). Beyond T-VEC, other OVs have demonstrated clinical promise. AdVs, like AdV-p53, when combined with chemotherapy and radiotherapy, achieved an 82% response rate in malignant pleural effusion patients receiving cisplatin (224, 225). In China, Oncorine (H101), a AdV, was approved for nasopharyngeal carcinoma, showing a response rate exceeding 78% when combined with chemotherapy (226). Our group has made significant contributions to OVT and vaccine development, particularly in genetic engineering. Inactivating the UL37 deamidase in the VC2 (an HSV-1 variant) enhanced viral replication, spread, and GM-CSF secretion, boosting its immunogenic potential (227). Preclinical studies show that the VC2 increases immune activation, converts immunosuppressive “cold” tumors into immunostimulatory “hot” tumors and improves survival in mouse models of melanoma and breast cancer (151, 227, 228). This underscores the importance of targeted viral modifications to optimize OV vaccines.

In addition, we have explored live-attenuated HSV vaccines for genital and ocular HSV infections, focusing on their ability to elicit robust immune responses. Preclinical studies are ongoing to advance these vaccines into human clinical trials (229). Notably, several HSV-based OVs have progressed into clinical testing. In a Phase 1b trial for recurrent GBM, G207, administered into resected tumor cavities, demonstrated a strong safety profile and significant CD8+ T-cell and macrophage infiltration. In a first-in-human trial for pediatric malignant pediatric cerebellar tumors, 11 out of 12 patients showed positive clinical responses, with four surviving beyond 18 months, significantly exceeding the median survival of 5.6 months with standard therapies (230).

Another novel oHSV, HSV1716, has been investigated for melanoma, mesothelioma, and solid tumors. In mesothelioma patients treated intrapleurally, Th1 cytokines (IL-2, IFN-γ, TNF-α) were elevated, and antitumoral antibody responses were detected. Intravenous HSV1716 was well tolerated in young patients with extracranial solid tumors, with some achieving stable disease (231). Similarly, HF10, an attenuated HSV-1, has shown safety and efficacy in pancreatic cancer, head and neck squamous cell carcinoma, melanoma, and breast cancer, increasing immune cell infiltration and inducing immune responses. Our group has also worked on other types of OVs, including those based on measles virus and VSV (1). A Phase I/II trial of MV-NIS (an engineered measles virus encoding the sodium iodide symporter) in ovarian, primary peritoneal, and fallopian tube cancer patients, showed promising results. The virus replicated, induced antitumor responses, and was well-tolerated, with some patients experiencing grade 3–4 adverse events, like neutropenia and leukopenia. One patient achieved a complete response that lasted for more than 10 years (232),.

Additionally, our team has also pioneered the development of Jurona virus (JURV), demonstrating its therapeutic potential in HCC. In preclinical models, JURV monotherapy led to significant tumor regression, while its combination with anti-PD-1 therapies improved survival outcomes by reshaping the immune landscape within the TME (14). The trivalent live-attenuated measles, mumps, and rubella (MMR) vaccine also have similar oncolytic applications (33). Other OVs under clinical evaluation include Pexa-Vec, a poxvirus-based OV tested in HCC. High-dose Pexa-Vec in combination with sorafenib extended median OS to 14.1 months compared to 6.7 months in the low-dose cohorts. However, the Phase III PHOCUS trial was discontinued due to insufficient survival benefits (233). In contrast, G47Δ, a third-generation HSV-1 derivative, has received conditional approval in Japan for GBM, demonstrating an 84.2% one-year survival rate and robust tumor-infiltrating CD4+/CD8+ T-cell responses (234). Reovirus-based therapy, particularly pelareorep, has shown tumor stabilization in 86% of metastatic cancer patients, although few have progressed beyond early-phase trials. The VacVDD, a VacV with double TK gene deletion, exhibits systemic tumor targeting in advanced solid tumors but shows a median survival of 4.8 months underscoring the need for further optimization (235). While OVs have shown promise in early-phase clinical trials, translating these results into consistent survival benefits in late-phase trials remain challenging. However, novel engineering strategies, such as arming OVs with ICIs, suicide genes, and tumor-specific promoters, continue to enhance their therapeutic potential. Our ongoing research into OV-TME interactions, in combination with established immunotherapies like CART cells and checkpoint blockade, represents a crucial step in improving OV efficacy and solidifying their role as mainstays in cancer immunotherapy (14, 227, 236).

## 6 New immune and metabolic classification of tumors to guide oncolytic virotherapy

To optimize the clinical application OVs, we propose a new classification system for OV-responsive tumors based on their immune, metabolic, and genetic landscapes. This framework is designed to guide OV selection and engineering therapeutic efficacy (**Table 2**).

**Table 2:** Classification Model for OV-Responsive Tumors: This model enables personalized OV therapies by aligning the tumor's unique features with optimized viral engineering strategies.

Tumor type	Examples	Key features	Best OV strategy	References
Immune Hot Tumors	Melanoma, NSCLC, TNBC, Bladder cancer (High tumor mutational burden, CIS, T-cell inflamed tumors)	High T-Cell infiltration, high PD-L1, responds well to immunotherapy	OV with checkpoint inhibitors (PD-L1, CTLA-4,), immune-stimulatory genes (GM-CSF, IL-12) + Atezolizumab + Nab Pacitaxel	(204, 239)
Immune Cold Tumors	Pancreatic adenocarcinoma, TNBC (ER+/HER2- subtype), Bladder cancer with non-muscle invasive and low tumor mutational burden, glioblastomas with highly immunosuppressive TME, low tumor mutational burden	High Tregs / MDSC density, low immune infiltration and activation, poor response to ICIs, requires combination approaches	OVs with STING agonist, cytokines, and TME-modifying enzymes + hormone therapy (e.g. Tamoxifen + Aromatase inhibitors) for ER+/HER2- subtype of breast cancer; Bacillus Calmette-Guerin (BCG) or chemotherapy for bladder cancer + Checkpoint inhibitors	(240)
Immune-Excluded Tumors	Colorectal cancer, prostate cancer (low tumor mutational burden, T-cell exclusion in adenocarcinomas subtype	T-cell Exclusion, Stromal Barriers, Vascular Abnormalities, fibroblast activation	OVs with matrix-degrading enzymes or VEGF blockade + androgen deprivation therapy, ICIs in MSI-high cases of prostate cancer	(2, 33)
Glycolytic Tumors	TBNC (HER2+ subtypes), Colorectal cancer, FGFR3-mutated bladder tumors, glioblastomas with Warburg Effect and IDH-wildtype glioblastoma	High lactate, low pH, hypoxic, tumor rely on high glucose metabolism, making them susceptible to metabolic inhibitors	OV with pH-resistant envelopes, metabolic inhibitors (LDH, P13K inhibitors) + metabolic inhibitors (e.g. metformin, GLUT1 inhibitors) for TBNC (HER2+ subtypes); or FGFR inhibitors (e.g. Erdafitinib) for FGFR3-mutated bladder tumors; or IDH inhibitors + metabolic targeting for glioblastomas	(124, 125, 207, 208)
Mutation-Driven Tumors	Glistoblastoma, TNBC (HER2+, TP53 mutations subtype); bladder cancer with high TMB subtypes, TP53, ERCC2 mutations; Glioblastomas with amplifications, PTEN loss, MGMT methylation	Defective p53, RAS, RB1, high tumor mutational burden, frequent DNA damage response defects	OVs engineered for synthetic lethality with tumor mutations with ICIs + HER2-targeted therapy (e.g. Trastuzumab and Pertuzumab) + Temozolomide + Radiation	(116, 237, 238)
Chromosomal Instability Tumors	Ovarian cancer	MSI aneuploidy, BRCA1/2 loss	OV with IFN-activating payloads, DNA repair inhibitors	(286)
Epigenetically Driven Tumors	Liver cancer, TNBC (ER+ subtype, BRACA1/2 mutant TNBC), epigenetically driven prostate cancer with AR-dependent pathways, BRCA1/2, EZH2 dysregulation, IDH1/2 mutant glioblastoma, with histone modifications	High DNA methylation, governed by histone modifications, and chromatin remodeling, often targetable by epigenetic drugs	OVs combined with epigenetic drugs, CRISPR-based reprogramming + PARP inhibitors (e.g. Olaparib) + HDAC inhibitors; or PARP inhibitors (Olaparib) + AR-targeted therapies or IDH inhibitors + HDAC inhibitors for glioblastomas	(287)

### 6.1 A new classification system for OV-responsive tumors

As the field of OVT advances toward precision medicine, there is a growing need for systematic frameworks that can stratify tumors based on their molecular characteristics. Recent studies have highlighted the importance of genomic and epigenomic alterations in shaping tumor susceptibility to OV infection, replication, and immune activation. This section introduces a molecular classification system that categorizes tumors according to their genetic, epigenetic, and immunologic profiles, providing a foundation for tailoring OVT strategies to individual tumor vulnerabilities. By aligning therapeutic design with tumor-intrinsic features, this approach aims to enhance treatment efficacy and expand the clinical applicability of OVT.

Tumors with specific genetic alterations, such as loss-of-function mutations in tumor suppressor genes like p53 or RB1, exhibit impaired antiviral responses, rendering them highly permissive to OV replication. For instance, GBM, frequently harboring these mutations, represent ideal candidates for OVs engineered to exploit such vulnerabilities, particularly when paired with synthetic lethal agents that further sensitize tumor cells to oncolysis (116, 237, 238). Tumors characterized by chromosomal instability, such as ovarian cancer, often display defective DNA repair mechanisms. These tumors are highly responsive to OVs armed with interferon-stimulatory transgenes, which trigger innate immune activation. The therapeutic efficacy of these OVs is further amplified when combined with ICIs, promoting durable antitumor immunity. This genetic-based classification system offers a structured framework for designing personalized OVT, optimizing treatment by targeting the unique immune, metabolic, and genetic traits of tumors. Ultimately this approach aims to improve patient outcomes and drive greater clinical success.

In contrast, epigenetically driven tumors, such as hepatocellular carcinoma, exhibit widespread DNA hypermethylation and histone modifications that repress immune-related gene expression. In such tumors, OV responsiveness can be enhanced through epigenetic reprogramming using hypomethylating agents or histone deacetylase inhibitors, which restore immune surveillance pathways. Moreover, CRISPR-based genome editing strategies offer promising avenues to augment OV replication and restore tumor suppressor activity, further improving therapeutic outcomes.

This molecularly guided classification system allows for precision-matched OVT, tailored to the genetic, metabolic, and immunologic profiles of individual tumors. By integrating OVs with co-therapies that address the specific vulnerabilities of each tumor subtype, this strategy holds the potential to revolutionize personalized cancer immunotherapy and significantly improve patient prognosis.

### 6.2 Immune-based classification: targeting tumor immune profiles

Tumors exhibit distinct immune profiles that influence OV efficacy. Immune-hot tumors, such as melanoma and non-small cell lung cancer, feature high T-cell infiltration and an inflamed TME, responding well to OVs, especially when combined with ICI and immune-stimulatory cytokines (204, 239). Immune-cold tumors, like pancreatic adenocarcinoma, have low T-cell infiltration and abundant immunosuppressive cells (240). OVs targeting these tumors should be engineered to reprogram the TME into an immune-permissive state, with combination therapies to modulate immune cell recruitment and activity (240). Immune-excluded tumors, such as colorectal cancer, present additional challenge as T cells is confined to the stroma, limiting tumor infiltration (33). OVs for these tumors should incorporate matrix-degrading enzymes to break down physical barriers, and VEGF blockade may further enhance penetration and therapeutic effectiveness (241).

### 6.3 Metabolism-based classification: targeting tumor metabolic vulnerabilities

Tumor metabolism significantly impacts OV replication and therapeutic efficacy. Glycolytic tumors, like TNBC, rely on aerobic glycolysis, creating an acidic, hypoxic environment (124, 125). OVs targeting these tumors should include pH-adaptive envelope proteins to survive in acidic conditions, with metabolic inhibitors to disrupt glycolytic pathways (207, 208). Oxidative tumors, such as ovarian cancer, primarily depend on oxidative phosphorylation and exhibit high ROS levels (187–190). OVs can exploit this oxidative stress to enhance replication, potentially combined with NADPH oxidase inhibitors to increase tumor susceptibility. Metabolically plastic tumors, like colorectal cancer, can switch between glycolysis and oxidative phosphorylation, complicating treatment. OVs targeting these tumors should disrupt metabolic flexibility, potentially combined with dual metabolic inhibitors to restrict the tumor’s ability to adapt to metabolic stress.

## 7 Future perspectives

While OVs have demonstrated notable promise, several biological, immunological, and delivery-related hurdles continue to limit their efficacy. Overcoming these challenges will be key to realizing their full therapeutic potential. Hence the future success of OVT depends on refining delivery methods, identifying predictive biomarkers, and combining OVs with other therapies to enhance efficacy and mitigate limitations.

### 7.1 Addressing delivery, immune, and personalization challenges in OV monotherapy

To overcome the dense ECMs, hypovascularity, and high interstitial fluid pressure that restrict intratumoral spread of OVs integration of tumor-penetrating peptides, enzymatic ECM degradation (e.g., hyaluronidase co-expression), and stromal remodeling agents to improve viral distribution within solid tumors, become pertinent (13). Nanoparticle- and hydrogel-based delivery platforms may also enhance OV retention and diffusion at tumor sites. immune cloaking techniques using polymer coatings or ex vivo OV-loaded immune cells, such as monocytes or DCs, to stealthily deliver the virus to tumors could be used to overcome pre-existing antiviral immunity in the host. Moreover, tumor-tropic mesenchymal stem cells (MSCs) have shown promise in shielding OVs from immune detection and improving delivery to immune-privileged sites like the brain. Sites such as the brain, eyes, and testes present natural obstacles to immune access. Nanoparticle-based delivery (for example encapsulating viruses in nanoliposomes), modular capsid engineering, and BBB-penetrating vectors can enhance OV access to these areas. Additionally, ultrasound-mediated delivery and convection-enhanced delivery are promising strategies to physically drive OVs into solid masses or across tight barriers (242, 243). To personalize OVT, robust biomarkers are needed to predict treatment response. Promising candidates include PD-L1 expression, tumor mutational burden, and deficiencies in antiviral pathways (e.g., STING, JAK) that facilitate OV replication. The development of imaging-based viral tracking, liquid biopsies, and RNA signatures may further guide patient selection and treatment monitoring.

### 7.2 Repositioning live vaccines as immunovirotherapy in cancer treatment and prevention

Live vaccines represent a groundbreaking approach in cancer immunovirotherapy, repurposing viral vaccines as powerful tools to combat cancer. Originally developed to prevent infectious diseases and some cancers, live vaccines are now being explored for their ability to activate both immune responses, directly targeting and eliminating cancer cells (243). This approach leverages the unique immunogenic properties of attenuated or engineered viruses, stimulating broad immune responses capable of targeting tumor cells throughout the body.

Oncolytic virus live vaccines are also used preventively to protect high-risk individuals from cancers driven by oncogenic pathogens. Unlike therapeutic live viruses, which are administered after malignancy develops, to convert tumors into *in situ* vaccines, preventive vaccines prime the immune system to recognize and eradicate cancer-causing infections before tumorigenesis occurs. They deliver viral antigens or virus-like particles that elicit neutralizing antibodies and durable memory responses. The HPV vaccine, which prevents cervical, anal, and oropharyngeal cancers by targeting high-risk HPV strains, and the hepatitis B virus (HBV) vaccine, which lowers hepatocellular carcinoma risk by preventing chronic HBV infection, are among the most successful examples. These interventions have demonstrated high efficacy in reducing infection rates and associated cancer incidence, underscoring their pivotal role in global cancer prevention strategies (244, 245).

However, the concept of prophylactic administration of OVs to prevent non-virus-induced cancers remains clinically unexploited. Also, in the absence of tumor tissue, OVs lack a target substrate for infection and lysis, precluding the release of TAAs necessary to prime an effective immune response. Furthermore, non-virus-driven malignancies do not share uniform pre-existing antigens that could be targeted in advance. Administering OVs to healthy individuals therefore poses safety risks without conferring the needed immunologic benefit. Consequently, OVs such as T-VEC and VV are reserved for therapeutic use in established cancers, where they convert tumors into *in situ* vaccines and synergize with other immunotherapies to drive robust antitumor immunity (244, 245).

Our group has investigated the potential of repurposing the live attenuated trivalent measles, mumps, and rubella (MMR) vaccine as a cost-effective cancer immunotherapy (33). In murine models of HCC and colorectal cancer, intratumoral MMR vaccine injections resulted in significant tumor growth inhibition and improved survival. The vaccine activated a robust CTL response and reduced TAMs, enhancing the antitumor immune response and offering a promising option for cancer immunotherapy. Additionally, the recombinant HSV-1 vaccine vector has shown promise for personalized cancer vaccines (151). A recombinant engineered oHSV-1 called VC2-OVA, expressing a fragment of ovalbumin fused with the VP26 viral capsid protein, demonstrated specific antitumor immunity in a murine melanoma model, significantly reducing tumor cell colonization in the lungs and extending survival (228). The effects of HSV-1 vaccine strain VC2 in a mouse model of stage four breast cancer was investigated, and VC2 treatment resulted in significant intratumor infiltration of functionally active T cells, inhibited tumor metastasis and reduced the expression of pro-tumor genes VEGF and PD-L1 (246). Similarly, intramuscular injection of mice with live attenuated VC2 vaccine strain protected against ocular challenges, which was associated with enhanced intracorneal infiltration of γδ T cells, which helped control ocular immunopathogenesis (246). These live vaccines mimic viral infections, stimulating immune responses that target tumor cells directly and generating systemic antitumor effects. They activate CTL, NK cells, and other immune effectors, which infiltrate the TME, promote tumor cell destruction and can address distant metastases. Notably, live vaccines can generate long-lasting immune memory, providing protection against tumor recurrence (233).

One of the key advantages of repurposing live vaccines for cancer is their ability to be readily available for cancer treatment without the need for extensive production or toxicology testing (247). Certain live vaccines, including those based on VSV, reoviruses, and engineered measles viruses, exhibit natural oncolytic activity. These viruses selectively infect and replicate within tumor cells, exploiting the unique characteristics of cancer, such as defective in antiviral responses and altered cellular machinery. This replication process causes tumor cell death, releasing viral particles that spread to adjacent cancer cells, amplifying the therapeutic effect.

In addition to their oncolytic properties, live vaccines also enhance immune responses against tumors that are typically resistant to immune attack. The viral infection itself acts as a “danger signal” that alerts the immune system to the presence of tumor cells, converting “cold” tumors (poorly infiltrated by immune cells) into “hot” tumors that are more receptive to immune-based therapies, including ICIs (248).

Several live vaccines are currently undergoing clinical trials for cancer treatment. The modified VacV used in the oncolytic vaccine Pexa-Vec has shown promise in treating melanoma and colorectal cancer. Similarly, engineered oncolytic measles viruses have demonstrated efficacy in tumor regression across multiple cancers, including GBM and ovarian cancer. The reovirus, with its selective oncolytic properties, has also shown potential when combined with chemotherapy and ICIs.

Despite these promising results, challenges remain, particularly concerning safety. Although engineered to minimize risks, live viral vectors may still pose potential threats, particularly for immunocompromised patients. However, OVs are often modified to attenuate their effects or selectively target cancer cells, reducing these risks. Rigorous monitoring during clinical application is crucial to detect and manage potential side effects, such as viral shedding or immune-related reactions (247).

One of the significant advantages of live vaccines is their ability to enhance other cancer therapies. When combined with ICIs, live vaccines have shown synergistic effects by boosting T-cell activity and improving immune cell infiltration, making tumors more susceptible to checkpoint blockade. Additionally, live vaccines can be combined with targeted therapies or chemotherapy, offering a multifaceted approach to overcoming tumor heterogeneity and resistance mechanisms.

Looking forward, live vaccines are poised to play an increasingly critical role in cancer treatment. As research deepens our understanding of immune responses and the TME, and as new viral vectors and delivery systems, e.g. are developed, live vaccine-based therapies will likely become a cornerstone of cancer immunotherapy. The potential of these vaccines to induce lasting immune responses, treat a broad range of malignancies, and prevent cancer recurrence offers new hope for patients facing challenging cancers. Ongoing research and clinical trials will continue to refine their applications, making live vaccine-based therapies a promising avenue for future cancer treatments.

### 7.3 Leveraging nutraceuticals to reprogram immunosuppressive cells and enhance OVs efficacy

Nutraceuticals, bioactive food-derived compounds, are emerging as promising adjuncts to OVT, particularly through their ability to reprogram immunosuppressive cells within the TME. By modulating immune pathways, these compounds can reduce populations of Tregs and MDSCs, shift TAMs from a pro-tumor M2 phenotype to an antitumor M1 phenotype, and enhance the infiltration and activity of CTLs and NK cells, all of which are critical for effective OVT. Curcumin, exhibit potent immunomodulatory and anti-inflammatory properties. It enhances both innate and adaptive immunity by activating CTLs and NK cells, inhibiting immunosuppressive cytokines and enzymes via NF-κB suppression, reprogramming M2-like TAMs into M1-like antitumor phenotypes, and reducing Treg and MDSC activity.

By modulating multiple biological pathways, nutraceuticals can improve immune responses, modify the TME, and increase cancer cells’ susceptibility to viral infection, thus improving the potency of OVT. Combining nutraceuticals with OVs holds great promise in optimizing treatment outcomes, overcoming resistance mechanisms, and improving patient survival (40). A primary mechanism through which nutraceuticals support OVs is by boosting the immune responses, as OVs rely heavily on the host’s immune system to effectively target and eliminate tumor cells.

Several nutraceuticals, including curcumin from turmeric, possess immunomodulatory properties that can activate immune cells such as CTLs and NK cells, key players in immune-mediated tumor destruction. Curcumin has been shown to enhance both innate and adaptive immunity by promoting immune cell activation, reducing immunosuppressive signaling or cytokines and enzymes by inhibiting NF-κB binding, and creating a more favorable environment for viral replication within the TME (256). Additionally, curcumin possesses anti-inflammatory and immunomodulatory qualities (249). Moreover, curcumin’s potential to enhance chemotherapy and ICIs suggests that it could increase the effectiveness of OVT. Curcumin has been shown to influence the proliferation and function of different immune cells, such as CTL and NK cells, potentially enhancing the immune system’s response to the tumor (249). Yet, curcumin might also suppress certain antiviral mechanisms that typically restrict OV replication. It can reduce the expression of ISGs, a critical component of the body’s antiviral defense (250). Furthermore, curcumin can trigger apoptosis in cancer cells, rendering them more vulnerable to OV-induced infection and destruction (251). Curcumin can inhibit angiogenesis within tumors, potentially starving them of nutrients and enhancing their vulnerability to OVT. Preclinical studies have shown that curcumin enhances cancer therapy (18), however, further clinical trials are needed to validate that curcumin could be a beneficial supplement to OVT, partly addressing some of the limitations of single use of OVs.

Other nutraceuticals, like resveratrol (from grapes and berries), epigallocatechin-3-gallate, a polyphenol in green tea), have both demonstrated the ability to boost NK cell activity and promote immune responses, thereby enhancing viral-induced tumor regression (18, 252). These compounds can improve OVT efficacy by modulating immune checkpoint molecules and promoting a more favorable immune environment within the TME.

In addition to modulating the immune system, nutraceuticals can help overcome TME barriers that limit OVT efficacy. The TME is often characterized by hypoxia, dense extracellular matrices, and immunosuppressive factors, all of which hinder viral replication and spread. Nutraceuticals, such as sulforaphane, found in cruciferous vegetables, can downregulate factors like VEGF and MMPs. However, upregulation of VEGF increases angiogenesis which facilitate the entry of oncolytic viruses like HSV-1, in cancer cells (253). Antioxidants such as vitamins C and E, known for reducing oxidative stress, help optimize the TME, promoting viral replication and immune function. These antioxidants also activate immune cells, fostering a more favorable environment and enhancing the overall efficacy of OVTs. Clinical trials exploring the use of vitamins C and E as adjuncts to cancer therapy have shown promising results, particularly in melanoma and head and neck cancers, indicating improved viral replication and immune response (254).

Nutraceuticals can also sensitize cancer cells to OVs by modulating cellular pathways critical for viral entry and replication. For example, quercetin, a flavonoid abundantly found in apples and onions, enhances oAdV therapy by influencing autophagy and apoptosis, which regulate cancer cells’ responses to viral infection (255). While autophagy facilitates viral particle degradation, apoptosis promotes viral-induced cell death. In liver cancer models, quercetin improved OVT efficacy, resulting in more effective tumor cell lysis (114). Similarly, berberine, an alkaloid derived from various herbs, has been shown to exhibit direct antiviral properties, complementing OV activity by enhancing viral replication and tumor cell killing (256). In combination with HSV-1, berberine has been shown to enhance viral replication and tumor cell killing in pancreatic and liver cancer models by modulating cellular stress responses and promoting viral entry (256).

Certain nutraceuticals also possess inherent antiviral properties that can boost OVT efficacy. For example, garlic extract, rich in sulfur compounds such as allicin, has demonstrated antiviral activity that supports viral replication and spread within tumors (257). In combination with oncolytic VSV, garlic extract could improve viral particle production and oncolytic activity in lung cancer models. By stimulating innate immune responses and inhibiting viral clearance, garlic extract can complement OV therapies. Omega-3 fatty acids from fish oil, known for their anti-inflammatory properties (257), can modify the TME to promote OV activity by suppressing inflammatory pathways like NF-κB and promoting resolution molecules such as resolvins and protectins, which reduce inflammation and boost OV efficacy (258).

Despite the promise of combining nutraceuticals with OVs, challenges remain in optimizing this strategy. The timing, dosage, and selection of nutraceuticals must be tailored to maximize their synergistic effects with OVs. Also, not all nutraceuticals are suitable for every cancer type or OVT. Moreover, patient-specific factors such as genetic background, tumor type, and disease stage must be considered to ensure the best therapeutic outcomes.

**Figure 4:**
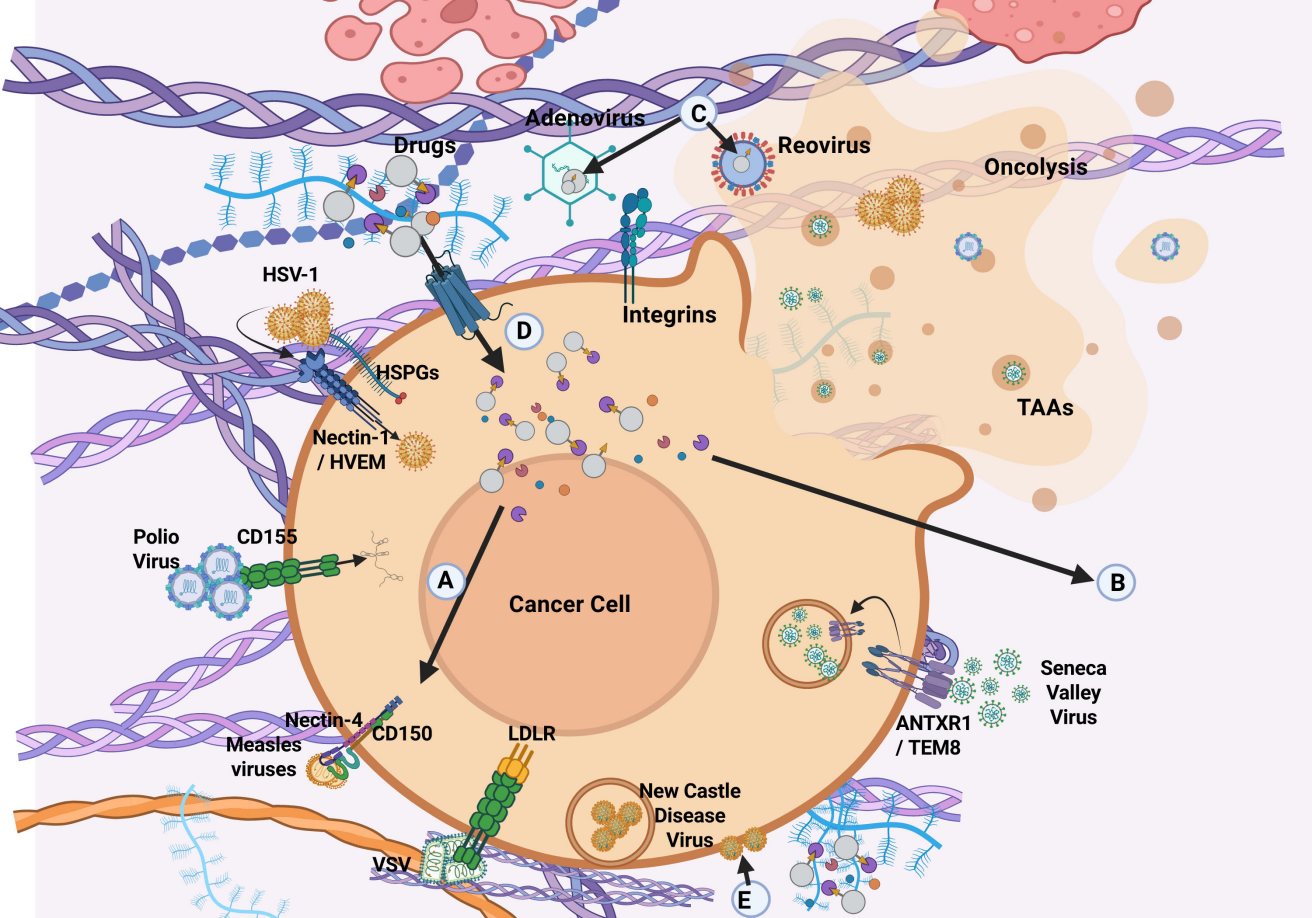
A proposed hypothesis for the pharmacological potentiation of OVT. HVEM = Herpesvirus Entry Mediator; CAR = Coxsackievirus and Adenovirus Receptor; ICAM-1 = Intercellular Adhesion Molecule 1; DAF = Decay-Accelerating Factor; SARs = Sialic Acid Receptors; SLAM = Signaling Lymphocytic Activation Molecule **(A)** Pharmacologically active natural or synthetic small molecules (drugs) can enhance the entry of OVs into solid tumors by upregulating the expression of viral entry receptors on the surface of cancer cells. **(B)** Certain therapeutic molecules promote vascular normalization within the TME, thereby facilitating endocytosis of viral particles and enhancing both oncolysis and immune modulation. **(C)** Biologically active small molecules can also be encapsulated within OV particles, enabling targeted delivery into the TME. **(D)** Upregulation of glucose or amino acid transporters on cancer cells supports increased uptake of biologically active compounds by cancer cells. **(E)** Some bioactive molecules, such as hyaluronidase and collagenase, disrupt the extracellular matrix or increase vascular permeability, enhancing OV penetration into tumor tissues (289).

Integrating nutraceuticals into OVT represents a novel approach to overcoming barriers in cancer treatment (**Figure 4**). By enhancing the immune responses, modifying the TME, and boosting viral replication and sensitivity, nutraceuticals and other small molecular scaffolds inspired from the latter can significantly amplify OVs efficacy. Ongoing preclinical and clinical studies continue to explore these combinations, with promising early results suggesting that nutraceuticals may overcome some limitations of monotherapy. Combining nutraceuticals with OVs is not without challenges. The timing, dosage, and selection of nutraceuticals must be tailored to maximize their synergistic effects with OVs. Also, not all nutraceuticals are suitable for every cancer type or OVT. Moreover, patient-specific factors such as genetic background, tumor type, and disease stage must be considered to ensure the best therapeutic outcomes. As research advances, this integrated approach has the potential to transform cancer therapy, providing a more robust and personalized treatment strategy for patients with hard-to-treat cancers.

## 8 Conclusion

OVT is an emerging field that works both locally and systemically, stimulating adaptive antitumor immunity. Small-molecule inhibitors derived from natural products or synthetic scaffolds targeting key signaling pathways are gaining momentum in immuno-oncology; however, their therapeutic impact as monotherapies has been modest, often requiring combination with other modalities to achieve durable clinical responses. Combining OVs with these inhibitors, guided by mechanistic insights, presents a promising strategy to enhance cancer treatment. Preclinically, small molecules boost OV efficacy. However, those inhibiting PI3K-AKT-mTOR and KRAS-ERK/MAPK oncogenic pathways may suppress cancer and immune cells alike, leading to unintended consequences, like impaired T-cell function, and weakened immune responses. Managing toxicity to normal cells is important, and treatment timing plays a key role. If cancer cells die too early, it can reduce OV replication. In HCC, administering Pexa-Vec before sorafenib improves outcomes, but reduces OV effectiveness when administered together.

Nutraceuticals, such as curcumin, resveratrol, green tea polyphenols, omega-3 fatty acids, vitamin D, and selenium, further enhance OVT by modulating immunity, improving TME, and promoting viral replication. These natural bioactives, along with their synthetic scaffolds, offer the potential to make cancer therapies more effective and less toxic. The synergy between OVs, small molecules, and nutraceuticals offers a dual approach to boost viral oncolysis and immune responses. Clinical trials will be pivotal in optimizing combinations and dosages, paving the way for personalized and innovative cancer treatments that improve outcomes and quality of life.
